# A hybrid algorithm of grey wolf optimizer and harris hawks optimization for solving global optimization problems with improved convergence performance

**DOI:** 10.1038/s41598-023-49754-2

**Published:** 2023-12-21

**Authors:** Binbin Tu, Fei Wang, Yan Huo, Xiaotian Wang

**Affiliations:** 1 College of Intelligent Science and Engineering, Shenyang University, Shenyang, China; 2 College of Information Engineering, Shenyang University, Shenyang, China

**Keywords:** Computational science, Computer science, Information technology, Scientific data

## Abstract

The grey wolf optimizer is an effective and well-known meta-heuristic algorithm, but it also has the weaknesses of insufficient population diversity, falling into local optimal solutions easily, and unsatisfactory convergence speed. Therefore, we propose a hybrid grey wolf optimizer (HGWO), based mainly on the exploitation phase of the harris hawk optimization. It also includes population initialization with Latin hypercube sampling, a nonlinear convergence factor with local perturbations, some extended exploration strategies. In HGWO, the grey wolves can have harris hawks-like flight capabilities during position updates, which greatly expands the search range and improves global searchability. By incorporating a greedy algorithm, grey wolves will relocate only if the new location is superior to the current one. This paper assesses the performance of the hybrid grey wolf optimizer (HGWO) by comparing it with other heuristic algorithms and enhanced schemes of the grey wolf optimizer. The evaluation is conducted using 23 classical benchmark test functions and CEC2020. The experimental results reveal that the HGWO algorithm performs well in terms of its global exploration ability, local exploitation ability, convergence speed, and convergence accuracy. Additionally, the enhanced algorithm demonstrates considerable advantages in solving engineering problems, thus substantiating its effectiveness and applicability.

## Introduction

Among the various stochastic optimization methods, population-based algorithms inspired by nature are widely acclaimed and preferred. These approaches replicate problem-solving strategies employed by living organisms, as they pursue survival, which is the ultimate objective for all living beings. In order to achieve this objective, organisms have continuously evolved and adapted in diverse ways. Consequently, seeking inspiration from nature, the most effective and longstanding optimizer on Earth, is a sensible approach.

Swarm intelligence algorithms are a class of nature-inspired algorithms based on group behavior, which make use of mutual collaboration and information exchange among individuals to search for optimal or near-optimal solutions in the solution space. These algorithms are flexible and robust, self-organising and adaptive, and are capable of handling multimodal, high-dimensional, nonlinear and uncertain problems as well as multi-objective optimization and dynamic optimization^1,2^. These algorithms primarily reflect the behavior and representation of certain social aspects of a group of animals and their evolutionary process. Furthermore, they are characterized by mathematical simplicity, independence from gradient methods, and minimal mathematical derivation. These algorithms approach the problem in a meta-heuristic manner, wherein optimization is performed stochastically and the optimization process initially provides stochastic solutions^3^. As a result, optimization is achieved through an iterative process. Moreover, these algorithms exhibit stochastic features that allow them to avoid settling for the best solution among neighbouring candidate solutions, instead extensively exploring the entire search space.

**Figure 1:**
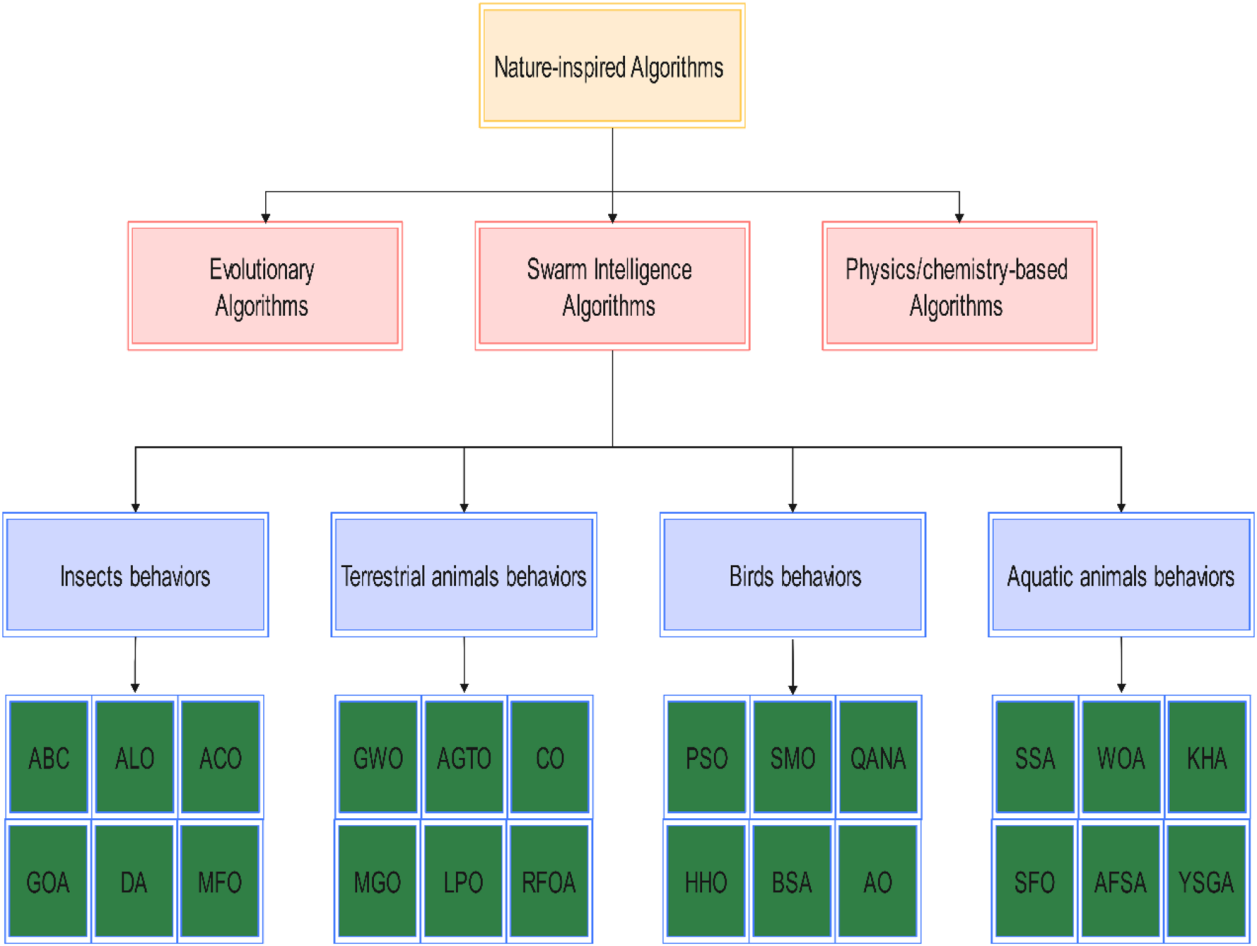
The classification of nature-inspired algorithms.

Currently, swarm intelligence algorithms have developed into a large family and been widely used by researchers, which are mainly inspired by animal behaviors such as migration, hunting, foraging, mating, nesting, and defending against enemies. It can be broadly classified into 4 types: insect-based, terrestrial animal-based, bird-based, and aquatic animal-based. Among these algorithms, some are well-known, including ant colony algorithm (ACO)^4^, particle swarm algorithm (PSO)^5^, grey wolf optimizer (GWO)^6^, whale optimization algorithm (WOA)^7^, ant lion optimizer (ALO)^8^, artificial bee colony algorithm (ABC)^9^, moth-flame optimization (MFO)^10^, Grasshopper optimization algorithm (GOA)^11^, dragonfly algorithm (DA)^12^, bat algorithm (BA)^13^, starling murmuration optimizer (SMO)^14^, salp swarm algorithm (SSA)^15^, african vulture optimization algorithm (AVOA)^16^, harris hawks optimization (HHO)^17^, aquila optimizer (AO)^18^, nutcracker optimizer (NOA)^19^, bird swarm algorithm (BSA)^20^, artificial gorilla troops optimizer (AGTO)^21^, quantum-based avian navigation optimizer algorithm (QANA)^22^, cheetah optimizer (CO)^23^, mountain gazelle optimizer (MGO)^24^, red fox optimization algorithm (RFOA), sailfish optimizer (SFO), krill herd algorithm (KHA)^25^, yellow saddle goatfish algorithm (YSGA) artificial fish swarm algorithm (AFSA)^26^, pride lion optimizer (LPO)^27^ and so on. The classification of the nature-inspired algorithms is showed in Fig. 1.

Nature-inspired algorithms include, in addition to swarm intelligence algorithms, other types of evolutionary-based, physics/chemistry-based, human-based, etc., which do not directly use cluster behavior in their algorithms. The most widely known of the evolutionary algorithms are genetic algorithms (GA)^28^, differential evolutionary (DE)^29^, evolutionary strategies (ES)^30^. Physics/chemistry-based algorithms are proposed based on mathematical models inspired by the laws of physics and chemistry, among which henry gas solubility optimization (HGSO)^31^, arithmetic optimization algorithm (AOA)^32^, etc. are cited frequently. Human-based algorithms often do not directly engage with nature; instead, they rely on factors such as human emotions, behavior, social interaction, and politics to develop systematic models. In recent years, social network search (SNS)^33^, political optimizer (PO)^34^, and anarchic society optimization (ASO)^35^ are representative algorithms.

The similarity between these types of nature-inspired algorithms lies in the fact that the solution kept being improved until it met the final criterion, and the optimization process would be divided the into two phases: exploration and exploitation^36^. Exploration refers to the tendency of algorithms with a high degree of stochastic behavior, so that solutions are subject to major changes. Larger variations in the solution will lead to greater exploration of the search space and thus to the discovery of its promising regions. However, due to the tendency of algorithms exploitation, smaller scale changes will happen, and solutions will tend to search locally. The global optimum of a specific optimization problem would be found by striking the proper balance between exploration and exploitation.

Among the huge number of swarm intelligence algorithms, grey wolf optimizer (GWO) proposed by Seyedali Mirjalili et al. in 2014 was inspired by the tracking, encircling, and hunting behaviors of grey wolf pack. GWO gets the advantages of fewer setup parameters being needed, easier to understand and implement, with faster convergence speed and higher solution accuracy. Moreover, GWO could be applied to engineering optimization^37,38^, economic load dispatch^39–42^, path planning^43–47^ and other fields. In no free lunch (NFL) theorem^48^, it logically proves that no single optimization technique could be utilized to solve all optimization problems. In other words, the average performance of algorithms in the field is equal while all optimization problems being considered. This theorem, to some extent, has contributed to the rapid growth in the number of algorithms proposed in the last decade. At the same time, there have been numerous performance improvement schemes for various algorithms for practical applications^49–54^, which is of course one of the motivations for this paper.

As a swarm intelligence algorithm proposed early, grey wolf optimizer also experienced the common issues which had been faced by many swarm intelligence algorithms, including limited global searchability and challenges about escaping from local optima. To address these issues, Mohammadet al.^37^ proposed one of the best improvements called I-GWO. This dimensional learning-based hunting (DLH) strategy enables each wolf to construct a neighborhood where neighboring information can be shared among wolf packs. Mahdis et al.^38^ introduced a search strategy called representative-based hunting (RH), which combines three efficient trial vectors inspired by the behavior of head wolves to improve population exploration and diversity. Mohammad et al.^55^ proposed a grey wolf optimizer called gaze cue learning based, benefiting from two new search strategies: neighbor gaze cues learning (NGCL) and random gaze cues learning (RGCL), inspired by the gaze cues behavior of wolves. NGCL enhances exploitation and the ability to avoid local optima, and RGCL improves the population diversity as well as the exploration–exploitation balance between exploration and exploitation. Wang et al.^56^ combined the principle of survival of the fittest (SOF) with differential evolution (DE) to iterate and eliminate the wolves with the lowest fitness values, while an equal number of new wolves being generated randomly. Ahmed et al.^57^ fused memory, evolutionary operators and stochastic local search techniques to the grey wolf optimizer, and further linear population size reduction (LPSR) techniques were integrated to achieve impressive performance on numerous benchmark functions and engineering optimization problems. Akbari et al.^58^ disregarded the social hierarchy in the GWO and randomly chose three agents to guide the population renewal mechanism. By incorporating a greedy algorithm, grey wolves will relocate only if the new location is superior to the current one. Shahrzadet al.^59^ added evolutionary population dynamics (EPD) to GWO, similar to the principle of survival of the fittest, where the main operation is to cause the worst search agent to perish after each iteration and randomly regenerate it around α、β and δ wolves, which helps to fully utilize the carryover of each search agent's information. Jagdish et al.^60^ used exploratory equations and opposition-based learning (OBL) to refine GWO, and it was validated with statistical, diversity, and convergence analyses for 23 classical benchmark test problems. Sharma et al.^61^ combined the exploitation and exploration capabilities of the cuckoo search and grey wolf optimizer respectively, achieving a balanced algorithm that performed effectively in high-dimensional problems. Singh et al.^62^ improved the utilization of particle swarm optimization by incorporating the exploration capabilities of the grey wolf optimizer, which generated a variant that caused the quality and stability of the solution to boost considerably over the original two. Chi et al.^63^ transferred the exploratory capabilities of aquila optimizer (AO) to the grey wolf to better balance between the two capabilities during iterations by choosing with random probability whether to make the wolf flight capable or not.

The main contributions of this paper are as follows.

1. Latin hypercube sampling is used to initialize the population trying to cover the whole solution space as much as possible and the global search performance of the optimization algorithm is improved.
2. The original linear reduction function in grey wolf optimizer is replaced by a nonlinear function with local perturbations which mimicked the actual hunting style, so that the exploration and exploitation capabilities of the algorithm could be balanced.
3. In an effort to mitigate the overreliance on the α wolf and avoid getting stuck in a local optimum, it is proposed to modify the position updates of grey wolves by considering the average position of all individuals rather than solely moving towards the three wolves with the lowest fitness values. Moreover, random selection using Lévy flights would be incorporated to enhance the exploration range.
4. Hybridize the grey wolf optimizer with the exploitation phase of the harris hawks optimization. Multiple active time-varying position updating strategies in the HHO algorithm give grey wolves harris hawks-like flight capabilities and a wide field of view. Combined with the greedy strategy, a more appropriate position is selected for the final update to further accelerate the convergence of the population.
5. The proposed algorithm's performance is assessed by evaluating it on 23 classical test functions and CEC2020. It is then compared with other algorithms and improvement schemes of grey wolf optimizer.
6. Four engineering design problems are used to evaluate the effectiveness of the proposed algorithm in solving real-world problems.

The subsequent sections of this paper are organized as follows: "Grey wolf optimizer" section provides a concise overview of the grey wolf optimizer. "Proposed HGWO" section introduces the HGWO algorithm. In "Experimental results and discussion" section, we conduct the relevant experiments and analyses. Additionally, "Real application of HGWO in engineering" section presents four classical engineering problems. Lastly, "Conclusions and future work" section concludes the entire paper by offering a summary and outlook.

## Grey wolf optimizer

The design of GWO is to simulate the social behavior of four different populations of grey wolves, namely $\alpha ,\beta ,\delta$ and $\omega$ wolves. The individual behaviors of these four types of wolves are influenced by social hierarchy, with *α* wolves owning the highest status, and the wolves' collective collaborative and communicative behaviors have a greater impact on the improvement and optimization of the algorithm. The GWO ascertains the best solution by considering every agent's position as a probable solution to the optimization predicament. At the same time, $\alpha ,\beta$ and $\delta$ wolves are regarded as the optimal, suboptimal, and more optimal solutions respectively. Therefore, the remaining wolves ($\omega$ wolves) are directed to the promising region. The specific explanation of GWO could be found in the Online Appendix.

## Proposed HGWO

### Latin hypercube sampling to initialize populations

Latin hypercube sampling (LHS) is a method of approximate random sampling from a multivariate parameter distribution proposed by Mckay et al.^64^ in 1979. Random sampling does not spread the sample well over the entire interval when the sample size is small. Unlike random sampling, Latin hypercube sampling has the advantages of uniform stratification and the possibility of obtaining sample values in the tails with fewer samples. The convergence speed and accuracy of the algorithm are affected to some extent by the uniformity of the initial population distribution. The initial population generated randomly cannot meet the requirements of distribution rationality and population diversity in the search space. However, Latin hypercube sampling itself has the characteristics of uniform stratification and equal probability sampling, which can generate variables covering the entire distribution space. Therefore, Latin hypercube sampling is used during algorithm initialization to cover the entire search space as much as possible, further increasing the diversity of the initial population and improving the optimal search performance^65–67^. The following is the steps of Latin hypercube sampling.

1. Suppose $N$ samples are drawn from a hypercube of dimension $D$, where $x_{i}^{j} \in [lb,ub]$ denotes the definition domain space; $i = 1,2,...,N$ denotes the sample individual; $j = 1,2,...,D$ represents the dimensionality of the sample individual.
2. Partition each dimension of the hypercube into $N$ subintervals, i.e., $lb^{j} = x_{1}^{j} \le x_{2}^{j} \le ... \le x_{N}^{j} = ub^{j}$, in the corresponding definition domain, and finally obtain $N^{D}$ small hypercubes.
3. Generate a fully aligned matrix with $N$ rows and $D$ columns.
4. Each row of the matrix $A_{N \times D}$ corresponds to a small hypercube, and then a sample is randomly selected from each small hypercube to obtain $N$ samples.

**Figure 2:**
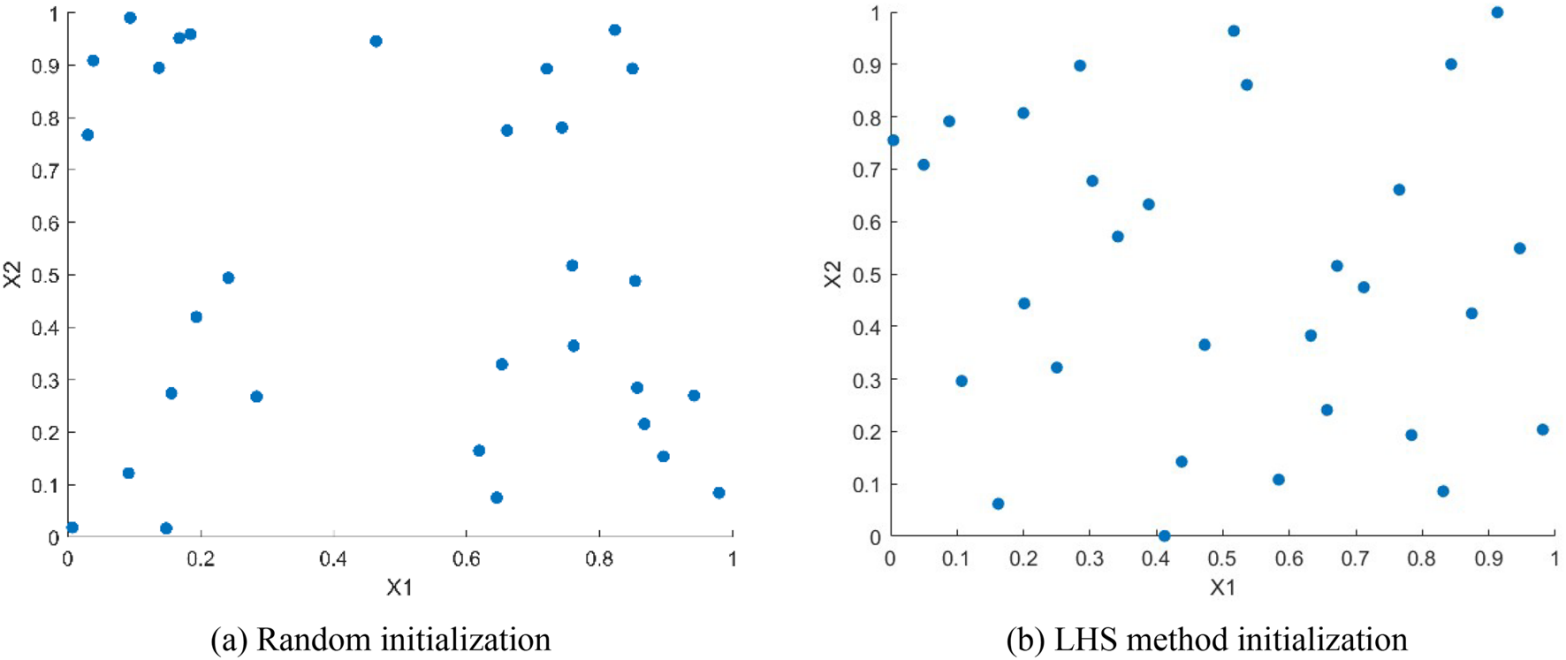
Two methods to initialize the population distribution.

In order to demonstrate the effectiveness of the Latin hypercube sampling initialization strategy visually, random initialization and Latin hypercube sampling initialization were adopted to initialize the grey wolf population through experiments respectively, and the population distribution maps is shown in Fig. 2.

In Fig. 2, when employing a stochastic approach for population initialization, the majority of individuals in the population are concentrated in the fringe region, resulting in reduced population diversity in the central region. Conversely, employing Latin hypercube sampling for population initialization yields a more uniform distribution of grey wolf individuals across the entire search space, thus showcasing the intuitive efficacy of the Latin hypercube sampling initialization strategy. Additionally, the experimental section of this article provides quantitative evidence supporting the effectiveness of the Latin hypercube sampling initialization strategy.

### Nonlinear convergence factors with local perturbations

**Figure 3:**
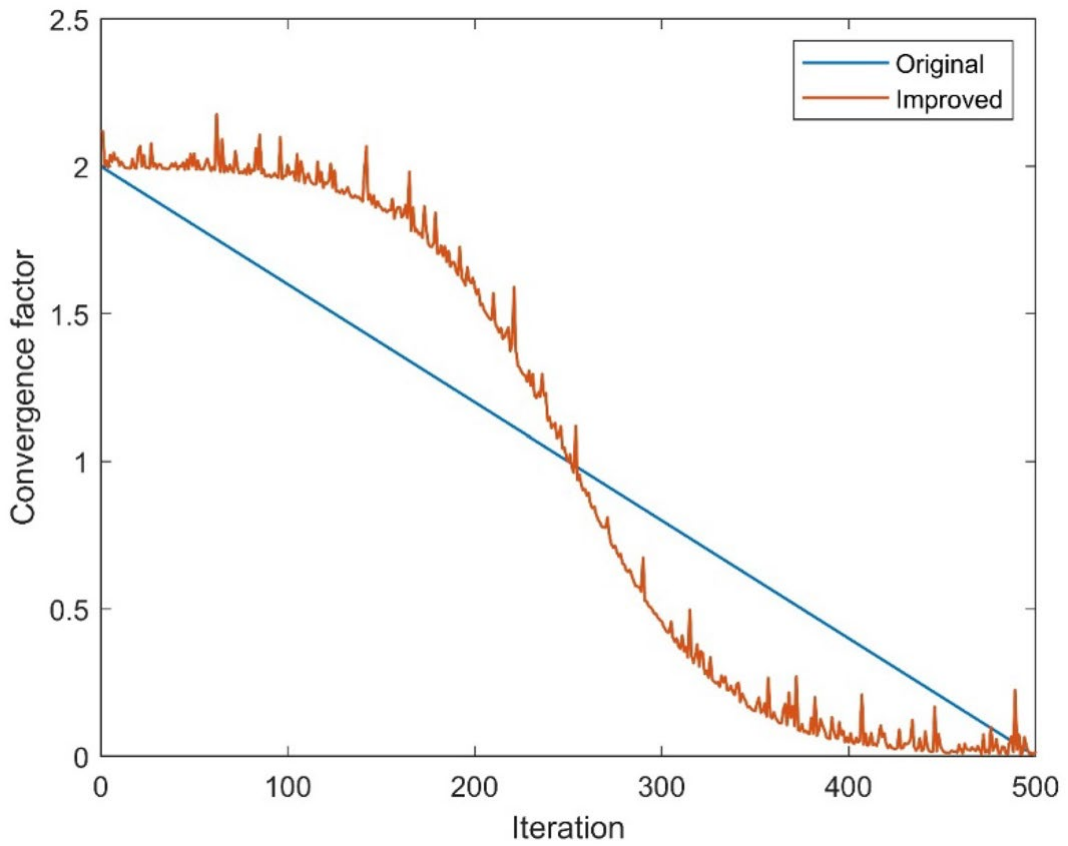
Comparison of two convergence factors.

In the initial grey wolf optimizer, the convergence factor linearly decreased from 2 to 0, and the change in convergence directly affected the algorithm's optimization ability, resulting in an imbalance in the algorithm's search ability before and after. Meanwhile, considering that the hunting behavior of grey wolves is analogized as a complex nonlinear process, this paper constructs a new type of nonlinear decreasing convergence factor based on the characteristics of the sigmoid function. Local perturbation of the convergence factor by introducing a random number $gamrnd$ that conforms to the gamma distribution improves the global search ability of the algorithm in later iterations and reduces the possibility of falling into a local optimum. The mathematical expression of the improved convergence factor is as follows.

1 $$ a = \frac{2}{{1 + e^{0.025(t - T/2)} }} + \varepsilon \cdot gamrnd $$

where $e$ is the base of the natural logarithm function; $\varepsilon$ is the perturbation adjustment coefficient, $\varepsilon$ value of 0.1 has the best effect after many tests; Fig. 3 shows the simulation graph of two different convergence factors for 500 iterations. It could be seen clearly that the value of improved convergence factor is larger and the decay speed is slower in the early stage, for the reason that the wolf has a strong global search ability to detect the upper and lower bounds in a wide range. In the middle, the value declined faster, which is conducive to the wolves moving fast towards the prey position to accelerate the convergence speed of the population. In the late stage, the value of $a$ is taken to be smaller and the speed of decreasing is relatively slow. At this time, the local search ability of the wolf pack is strengthened, and the fine search would be carried out in a small area close to the prey to reduce the omission of the solution. As a consequence, the nonlinear convergence factor proposed in this paper could balance the global search ability and local search ability more effectively than in original grey wolf optimizer.

### Expanded exploration

In the grey wolf optimizer, wolves move with complete inclination to the positions of α、β and δ wolves, and have limited ability to cope when caught in a local optimum. Accordingly, a position update formula for extended exploration is proposed.

2 $$ X(t + 1)^{\prime } = \left( {X_{1} + X_{2} + X_{3} } \right)/3 + X_{M} (t) - r \cdot X_{\alpha } (t) $$

3 $$ X_{M} (t) = \frac{1}{N}\sum\limits_{{i{ = 1}}}^{N} {X_{i} } (t) $$

where $X_{M} (t)$ denotes the mean position vector of all grey wolves at the $t$ iteration; $r$ takes a random vectors in the interval [0,1]. The position update formula proposed in this paper modifies the original formula by diminishing the absolute influence of α, β, and δ wolves on individuals while introducing mutual attraction among individuals. Instead of moving towards the three wolves with the lowest fitness value, the formula replaces this strategy. Furthermore, it reduces the random influence of the α wolf in position updating. As a result, the grey wolf population avoids excessive dependence and being trapped in local optima, particularly when the fitness value of the α wolf is notably lower than that of the β and δ wolves.

The Lévy flight function is a randomized wandering algorithm that simulates a random flight process and is widely validated for improving optimization problems^68–71^. The Lévy flight function embodies a randomized search algorithm, which effectively approaches the optimal solution by traversing in a randomized and meandering manner, enabling efficient global search. To mitigate the limitations of this algorithm in escaping local optima and to increase the probability of discovering the optimal solution, this paper introduced a novel position update formula that facilitates extended exploration in tandem with the Lévy flight.

4 $$ X(t + 1)\prime = k \cdot X_{\alpha } (t) \otimes levy $$

5 $$ k = \lambda (X(t) - X_{\alpha } (t)) $$

6 $$ levy = \frac{u}{{|v|^{1/\beta } }} $$

7 $$ \sigma_{u} = \left\{ {\frac{{{\Gamma }(1 + \beta )\sin (\beta \pi /2)}}{{{\Gamma }((1 + \beta )/2)2^{(\beta - 1)/2} \beta }}} \right\}^{1/\beta } $$

8 $$ \sigma_{v} = 1 $$

where $\otimes$ is the dot product operator; $k$ is the step control parameter; $\lambda$ takes the value of constant 0.01; $u$,$v$ satisfy the normal distribution, $u \sim N(0,\sigma_{u}^{2} )$, $v \sim N(0,\sigma_{v}^{2} )$; usually $\beta$ takes the value between [0,2], in this paper, $\beta = 2R$, $R$ is the random numbers between [0,2].

9 $$ \left\{ \begin{gathered} X(t + 1)^{\prime} = \left( {X_{1} + X_{2} + X_{3} } \right)/3 + X_{M} (t) - r \cdot X_{\alpha } (t)\;\;\;\;\;\;c < 0.5 \hfill \\ X(t + 1)^{\prime} = k \cdot X_{\alpha } (t) \otimes levy\;\;\;\;\;\;\;\;\;\;\;\;\;\;\;\;\;\;\;\;\;\;\;\;\;\;\;\;\;\;\;\;\;\;\;\;\;c \ge 0.5 \hfill \\ \end{gathered} \right. $$

The selection of the two aforementioned position update formulas is based on a randomly sampled number $c$ from the interval [0, 1]. During the early search phase, when the second position update formula is selected with a certain probability, the large step size allows for an expansion of the search scope and promotes exploration and discovery. This is beneficial for enhancing the diversity of the population and significantly reduces the risk of the grey wolf algorithm getting trapped in local optima. In the later search phase, when the global optimal solution range has been largely determined, the first position update formula continues to attempt to escape from local optima and improve the solution quality.

### Location update based on HHO

The position update of the grey wolf optimizer is guided by the three most adaptive wolves, although they are unaware of the exact location of the prey. Consequently, this search method is susceptible to converging to local optima. The harris hawks optimization (HHO)^17^ is a novel, population-based, nature-inspired optimization technique that draws inspiration from the cooperative behavior of harris hawks in nature, specifically their surprise attacks during the chase. HHO incorporates multiple and time-varying position update strategies that contribute to its comparatively fast convergence speed. The hybridization of GWO and HHO can improve GWO's convergence rate and enhance search accuracy. When the exploitation strategies of HHO are combined with GWO, they form a more complex and effective cooperation mechanism. By leveraging HHO's methods for information exchange, local search, and global search, wolves in the GWO can cooperate and coordinate more effectively, thus accelerating the search process and increasing search diversity.

Harris hawks can adjust their behavior based on the prey's escape energy level. During the prey's escape process, its energy $E$ will significantly diminish.

10 $$ E = 2E_{0} \left( {1 - t/T} \right) $$

In view of the difference in escape energy between different prey animals, $E_{0}$ (initial value of escape energy) is randomly changed within [− 1,1] in iteration process of the algorithm.

Given the differences in escape energy between prey, the $E_{0}$ (initial value of escape energy) is made to vary randomly within [− 1, 1] during the iteration of the algorithm. When $\left| E \right| \ge 1$, HHO's exploitation strategy will not be performed in HGWO, but rather the exploration area should be expanded as much as possible and solution omissions should be reduced. With the prerequisite of $\left| E \right| < 1$ fulfilled, and the position of the prey replaced by that of the α wolf, the harris hawks begin to make raids on their prey. Unfortunately, the prey often escaped one step ahead of the harris hawks. Thus, based on the prey's escape behavior and its own pursuit strategy, the Harris's hawk evolved four attack strategies. Suppose $\eta$ represents the escape probability of the prey, which is a random number between [0,1], with a successful escape when $\eta < 0.5$ and a failed escape when the opposite is true.

#### Soft besiege

When $\eta \ge 0.5$ and $\left| E \right| \ge 0.5$, the quarry retains ample vigor to attempt to elude the pursuit through evasive hops. In this case, the current position update can be done using Eq. (11).

11 $$ X(t + 1)^{\prime\prime} = X_{\alpha } (t) - X(t) - E\left| {J \cdot X_{\alpha } (t) - X(t)} \right| $$

12 $$ J = {2} \cdot (1 - R) $$

where $R$ is a random number within the range [0,1] and determines the magnitude of the random leaps of the prey during the process of escaping.

#### Hard besiege

When $\eta \ge 0.5$ and $\left| E \right| < 0.5$, the prey is in a state of exhaustion, facing no choice but heading to grab the ground. At this time, harris hawks overpower directly, and finally perform the surprise attack.

13 $$ X(t + 1)^{\prime\prime} = X_{{\alpha { }}} (t) - E\left| {X_{{\alpha { }}} (t) - X(t)} \right| $$

#### Soft besiege with progressive rapid dives

When $\eta < 0.5$ and $\left| E \right| \ge 0.5$, prey has sufficient escape energy, then there is a chance to escape. In this situation, harris hawks engage in a careful strategy of gaining momentum through rapid dives gradually before launching an attack.

14 $$ Y = X_{\alpha } (t) - E\left| {J \cdot X_{\alpha } (t) - X(t)} \right| $$

After this position update, if the fitness value does not improve, another strategy would be executed, where $s = rand(1,D)$.

15 $$ Z = Y + s \cdot \lambda \otimes levy $$

In conclusion, the siege method can be outlined as follows.

16 $$ X(t + 1)^{\prime \prime } = \left\{ {\begin{array}{*{20}l} Y \hfill & {fitness(Y) < fitness(X(t))} \hfill \\ Z \hfill & {fitness(Z) < fitness(X(t))} \hfill \\ \end{array} } \right. $$

#### Hard besiege with progressive rapid dives

When $\eta < 0.5$ and $\left| E \right| < 0.5$, the prey doesn't have enough energy, so harris hawks will conduct an aggressive siege involving successive rapid dives with the purpose of closing the gap between themselves and prey.

17 $$ Y = X_{{\alpha { }}} (t) - E\left| {J \cdot X_{\alpha } (t) - X_{M} (t)} \right| $$

18 $$ Z = Y + s \cdot \lambda \otimes levy $$

Similarly, this type of siege can be summarized as shown in Eq. (19).

19 $$ X(t + 1)^{\prime \prime } = \left\{ {\begin{array}{*{20}l} Y \hfill & {fitness(Y) < fitness(X(t))} \hfill \\ Z \hfill & {fitness(Z) < fitness(X(t))} \hfill \\ \end{array} } \right. $$

The convergence of the algorithm will be further accelerated via the idea of greedy strategy through various position updating formulas mentioned in "Expanded exploration" and "Location update based on HHO" sections of this paper, and the final position updating formula is shown as below.

20 $$ X(t + 1) = \left\{ {\begin{array}{*{20}l} {X(t + 1)^{\prime } } \hfill & {fitness(X(t + 1)^{\prime } ) < fitness(X(t))} \hfill \\ {X(t + 1)^{\prime \prime } } \hfill & {fitness(X(t + 1)^{\prime \prime } ) < fitness(X(t))} \hfill \\ \end{array} } \right. $$

### Pseudocode of HGWO

**Figure 4:**
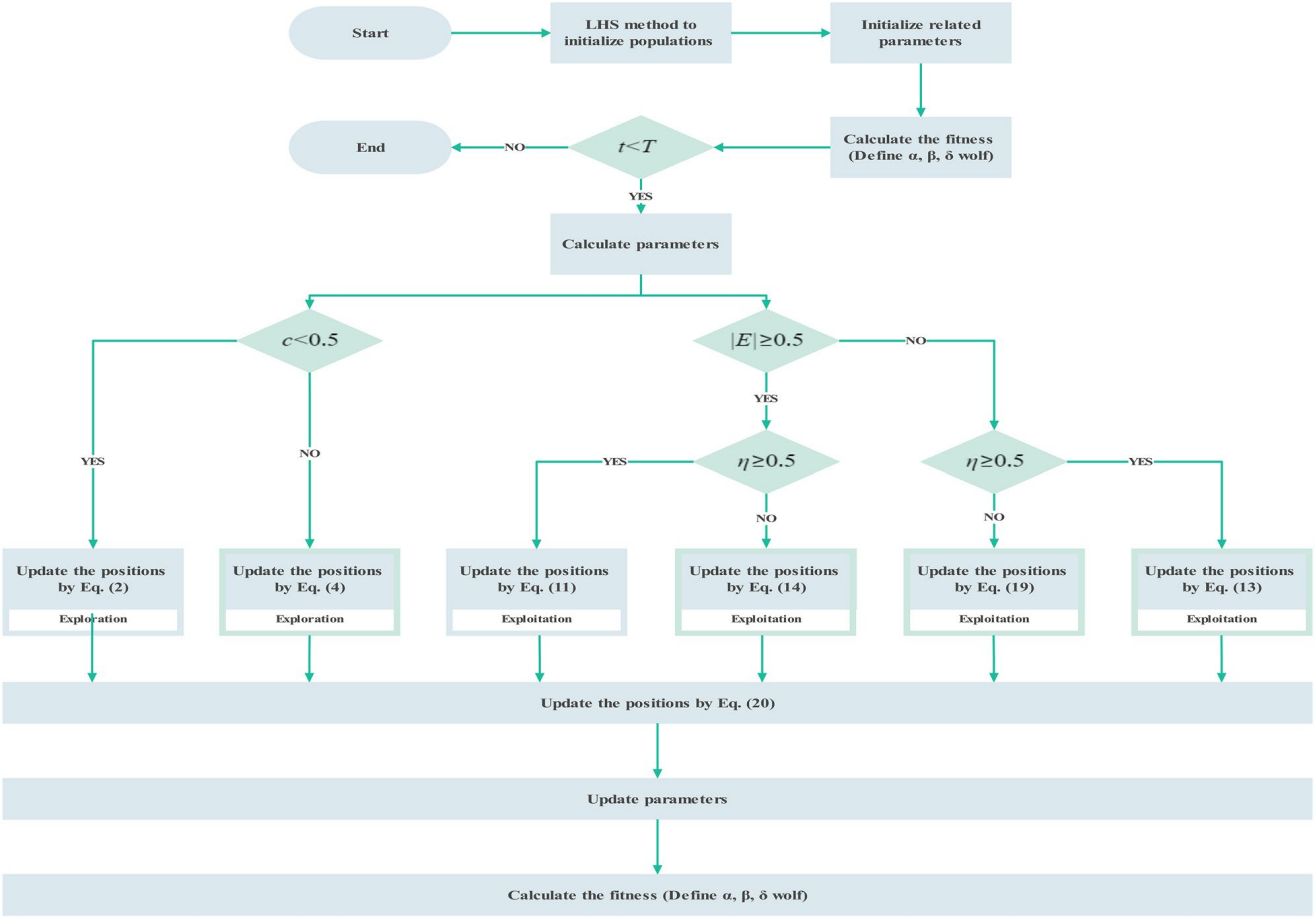
Flow chart of HGWO.

The whole algorithm will be performed iteratively through the above processes until being iterated to the preset maximum number. The flowchart of HGWO in Fig. 4 as well as the pseudocode of Algorithm 1 could be referred to broadly.

**Algorithm 1.**
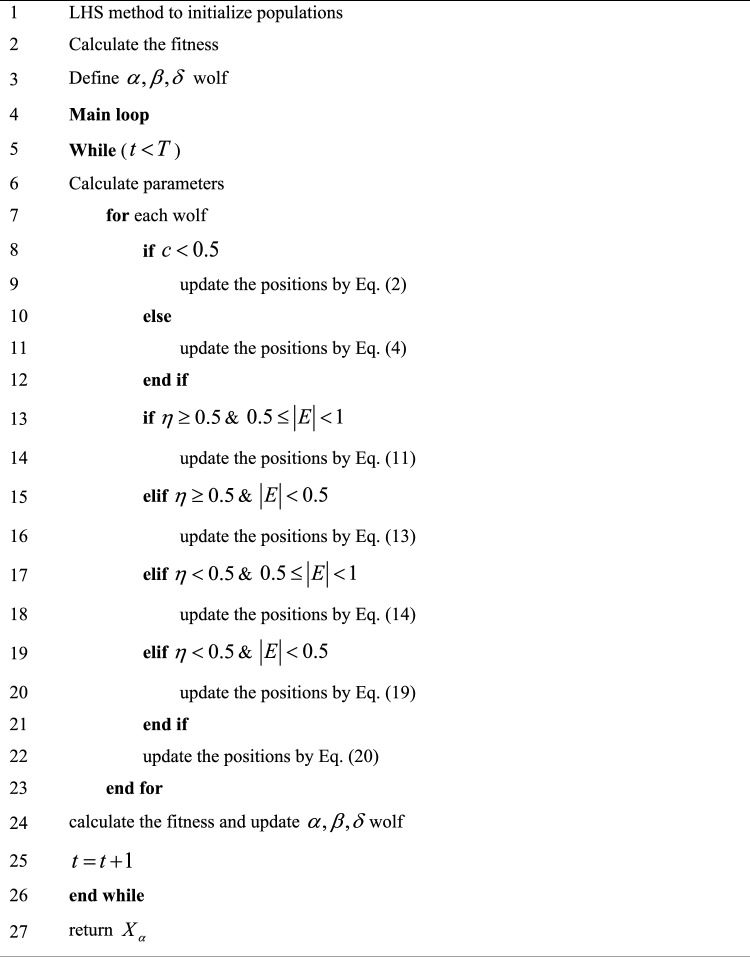
HGWO

## Experimental results and discussion

### Experimental setup

In the field of stochastic optimization, it is common to quantitatively evaluate the performance of different algorithms using a set of mathematical test functions that have known optimal values. However, it is important to vary the characteristics of the test functions in order to draw reliable conclusions. In this study, we will assess the performance of HGWO on 23 classical benchmark test functions commonly used in literature to measure the exploration and exploitation capabilities of newly proposed, improved algorithms^72–74^. Detailed information about these test functions, including their formulation, dimensionality (Dim), search space restriction (Range), and optimal reported value (Fmin), can be found in the Online Appendix. Furthermore, this study validates the performance of HGWO using the latest and challenging numerical optimization competition test suite: CEC2020^75–77^. This suite consists of 10 test functions that cover a range of characteristics, including unimodal, multimodal, hybrid, and combinatorial, and are specifically designed to challenge both previously proposed and newly proposed metaheuristic algorithms. The characteristics of these test suites are described in the Online Appendix.

To ensure the fairness and reasonableness of the simulation experiments, all algorithms in this paper have a population size of 30 and a maximum number of iterations set at 500. The simulations are conducted on a computer system consisting of an Intel Core i5-6300HQ CPU with a clock speed of 3.20 GHz, 12.0 GB of RAM, and Windows 10 Professional. The simulation software used is MATLAB R2022a. Each test function is solved 20 times to generate statistical results. The algorithms are then compared using various performance metrics, including the mean and standard deviation of the best solution obtained in the final iteration. Smaller values of these metrics indicate better algorithmic performance in terms of avoiding local solutions and identifying the global optimum. Qualitative results, such as convergence curves, grey wolf trajectories, search history, and average fitness of the population, are described and analyzed subsequently.

### Compare results using 23 classic test functions

#### Comparison with standard optimization algorithms

The performance of several optimization algorithms is compared in this paper by solving the 23 benchmark functions mentioned above. The algorithms considered include the standard grey wolf optimizer (GWO)^6^, whale optimization algorithm (WOA)^7^, golden jackal optimization (GJO)^78^, multi-verse optimizer (MVO)^79^, salp swarm algorithm (SSA)^15^, seagull optimization algorithm (SOA)^80^, arithmetic optimization algorithm (AOA)^32^, and the HGWO proposed in this paper. The parameter configurations for each algorithm were obtained from their respective sources, and the specific settings can be found in Table 1.

**Table 1:** Specific parameterisation of algorithms.

Algorithm	Parameter	Value
GWO (2014)	Convergence constant a	Linear reduction from 2 to 0
WOA (2016)	Convergence constant a	Linear reduction from 2 to 0
	b	1
MVO (2016)	Wormhole existence prob	[0.2, 1]
	Travelling distance rate	[0.6, 1]
SSA (2017)	Position update probability	0.5
AOA (2021)	Sensitivity α	5
	Acceleration	[0.2,1]
	Mu	0.499
SOA (2018)	Convergence constant F_C_	Linear reduction from 2 to 0
GJO (2022)	E_1_	Linear reduction from 1.5 to 0
	E_0_	[− 1,1]
AGWO_CS (2021)	Convergence constant a	Nonlinear reduction from 2 to 1
PSOGWO (2017)	Convergence constant a	Linear reduction from 2 to 0
	C_1&_ C_2_& C_3_	0.5
	w	[0.5,1]
AGWO (2022)	Convergence constant a	Nonlinear reduction from 2 to 0
	B	0.8

**Figure 5:**
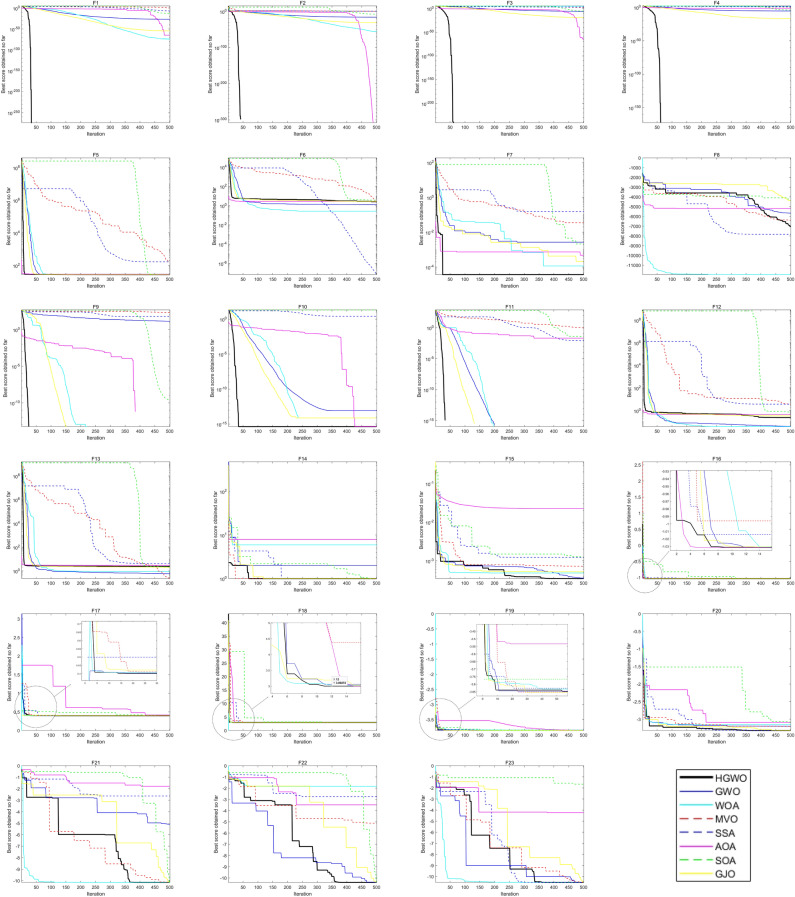
Convergence curve of the standard algorithms.

The optimal values, mean values, and standard deviations obtained are presented in Tables 2 and 3, with the best values highlighted in bold for easy comparison. The comparison of the results by the number of wins (W), ties (T), and losses (L) for each algorithm is shown at the end of each table. Moreover, the convergence curves of each algorithm on the benchmark functions can be observed in Fig. 5.

**Table 2:** Results of F1–F13 (Dim = 30).

	HGWO	GWO	WOA	MVO	SSA	AOA	SOA	GJO	AGWO_CS	PSOGWO	AGWO
F1											
Best	**0.00E + 00**	7.29E−29	1.20E−82	7.12E−01	2.96E−08	1.91E−127	0.00E + 00	1.58E−57	1.38E−47	5.77E−28	1.54E−155
Ave	**0.00E + 00**	1.69E−27	1.82E−70	1.30E + 00	1.58E−07	9.78E−23	6.77E−240	1.20E−54	8.90E−45	4.67E + 03	1.95E−147
Std	**0.00E + 00**	2.94E−27	8.15E−70	3.21E−01	1.50E−07	4.37E−22	0.00E + 00	2.11E−54	1.37E−44	9.85E + 03	7.45E−147
F2											
Best	**0.00E + 00**	1.69E−17	1.12E−56	4.07E−01	5.99E−01	**0.00E + 00**	6.15E−225	9.76E−34	2.76E−28	5.41E−15	4.62E−86
Ave	**0.00E + 00**	1.11E−16	1.40E−52	3.86E + 00	2.26E + 00	**0.00E + 00**	6.34E−173	7.29E−33	2.86E−27	6.54E + 06	1.21E−82
Std	**0.00E + 00**	6.52E−17	4.30E−52	1.30E + 01	1.14E + 00	**0.00E + 00**	0.00E + 00	6.73E−33	3.54E−27	2.84E + 07	3.25E−82
F3											
Best	**0.00E + 00**	1.05E−08	1.54E + 04	4.54E + 01	7.27E + 02	2.54E−110	1.34E + 04	4.30E−24	5.16E−15	9.84E−09	5.39E−93
Ave	**0.00E + 00**	1.18E−05	4.23E + 04	2.28E + 02	1.75E + 03	5.43E−03	4.16E + 04	1.37E−16	7.90E−10	8.66E + 03	2.81E−79
Std	**0.00E + 00**	3.57E−05	1.22E + 04	9.53E + 01	9.80E + 02	9.56E−03	2.79E + 04	5.60E−16	3.16E−09	1.90E + 04	1.24E−78
F4											
Best	**0.00E + 00**	9.91E−08	2.15E−01	7.58E−01	5.15E + 00	4.54E−77	1.52E−69	3.38E−18	1.23E−13	1.76E−08	1.16E−64
Ave	**0.00E + 00**	1.01E−06	3.99E + 01	1.99E + 00	1.17E + 01	2.73E−02	4.21E−15	1.08E−15	5.25E−12	4.09E + 00	5.37E−61
Std	**0.00E + 00**	1.47E−06	2.51E + 01	7.11E−01	3.21E + 00	1.59E−02	1.87E−14	3.08E−15	4.76E−12	1.46E + 01	8.76E−61
F5											
Best	2.64E + 01	**2.61E + 01**	2.68E + 01	3.94E + 01	2.73E + 01	2.79E + 01	2.87E + 01	2.71E + 01	**2.61E + 01**	2.62E + 01	2.72E + 01
Ave	2.81E + 01	2.72E + 01	2.81E + 01	2.82E + 02	2.74E + 02	2.84E + 01	2.88E + 01	2.78E + 01	**2.71E + 01**	1.05E + 04	2.76E + 01
Std	6.70E−01	6.24E−01	5.45E−01	5.78E + 02	5.43E + 02	2.31E−01	**3.76E−02**	6.60E−01	6.01E−01	3.56E + 04	5.71E−01
F6											
Best	2.00E + 00	7.11E−05	5.53E−02	6.15E−01	**3.24E−08**	2.56E + 00	1.27E + 00	1.50E + 00	7.50E−01	1.96E−03	2.50E + 00
Ave	3.25E + 00	7.63E−01	3.93E−01	1.21E + 00	**1.08E−06**	3.16E + 00	2.79E + 00	2.61E + 00	1.38E + 00	3.09E + 03	3.27E + 00
Std	5.90E−01	3.89E−01	2.41E−01	3.92E−01	**3.37E−06**	2.71E−01	9.28E−01	5.00E−01	3.25E−01	1.02E + 04	4.58E−01
F7											
Best	**3.45E−06**	4.52E−04	2.92E−04	2.00E−02	7.24E−02	4.18E−06	8.38E−04	6.00E−05	2.95E−04	5.32E−04	6.35E−05
Ave	**7.00E−05**	1.85E−03	3.12E−03	3.60E−02	1.53E−01	7.03E−05	3.98E−03	4.60E−04	1.50E−03	6.87E−02	2.41E−04
Std	**6.46E−05**	9.97E−04	3.00E−03	9.77E−03	7.07E−02	9.46E−05	3.86E−03	3.51E−04	1.48E−03	2.57E−01	1.77E−04
F8											
Best	− 6.82E + 03	− 7.40E + 03	− **1.26E + 04**	− 8.84E + 03	− 8.97E + 03	− 6.14E + 03	− 6.25E + 03	− 5.86E + 03	− 8.49E + 03	− 7.98E + 03	− 3.82E + 03
Ave	− 5.92E + 03	− 6.11E + 03	− **1.05E + 04**	− 7.69E + 03	− 7.53E + 03	− 5.36E + 03	− 5.19E + 03	− 3.66E + 03	− 6.86E + 03	− 6.77E + 03	− 3.19E + 03
Std	4.15E + 02	5.95E + 02	1.81E + 03	5.95E + 02	5.89E + 02	**3.36E + 02**	4.63E + 02	9.14E + 02	7.61E + 02	7.66E + 02	3.52E + 02
F9											
Best	**0.00E + 00**	**0.00E + 00**	**0.00E + 00**	6.77E + 01	2.59E + 01	**0.00E + 00**	6.82E−13	**0.00E + 00**	**0.00E + 00**	5.68E−14	**0.00E + 00**
Ave	**0.00E + 00**	4.10E + 00	2.84E−15	1.24E + 02	5.96E + 01	**0.00E + 00**	2.86E + 00	**0.00E + 00**	5.18E + 00	2.80E + 01	**0.00E + 00**
Std	**0.00E + 00**	5.87E + 00	1.27E−14	3.63E + 01	1.83E + 01	**0.00E + 00**	6.83E + 00	**0.00E + 00**	2.19E + 01	5.91E + 01	**0.00E + 00**
F10											
Best	**4.44E−16**	7.51E−14	**4.44E−16**	4.94E−01	1.65E + 00	**4.44E−16**	**4.44E−16**	4.00E−15	7.55E−15	1.18E−13	4.00E−15
Ave	**4.44E−16**	1.04E−13	3.29E−15	2.02E + 00	2.79E + 00	**4.44E−16**	**4.44E−16**	7.19E−15	8.26E−15	3.11E + 00	4.35E−15
Std	**0.00E + 00**	1.98E−14	2.47E−15	7.18E−01	8.85E−01	**0.00E + 00**	**0.00E + 00**	1.09E−15	1.86E−15	6.09E + 00	1.09E−15
F11											
Best	**0.00E + 00**	**0.00E + 00**	**0.00E + 00**	6.69E−01	1.02E−03	1.70E−02	**0.00E + 00**	**0.00E + 00**	**0.00E + 00**	**0.00E + 00**	**0.00E + 00**
Ave	**0.00E + 00**	4.56E−03	**0.00E + 00**	8.51E−01	1.52E−02	1.90E−01	**0.00E + 00**	**0.00E + 00**	**0.00E + 00**	2.53E + 01	**0.00E + 00**
Std	**0.00E + 00**	1.07E−02	**0.00E + 00**	8.05E−02	1.13E−02	1.41E−01	**0.00E + 00**	**0.00E + 00**	**0.00E + 00**	7.72E + 01	**0.00E + 00**
F12											
Best	7.31E−02	1.14E−02	5.05E−03	8.69E−01	2.87E + 00	**4.19E−03**	8.58E−02	6.16E−02	5.44E−02	2.04E−03	1.85E−01
Ave	2.89E−01	4.63E−02	**1.55E−02**	2.38E + 00	7.59E + 00	5.33E−01	4.02E−01	2.24E−01	1.03E−01	1.11E + 07	2.68E−01
Std	1.10E−01	3.31E−02	1.28E−02	1.07E + 00	3.19E + 00	**1.13E−02**	2.41E−01	7.54E−02	3.88E−02	4.95E + 07	5.80E−02
F13											
Best	**2.12E−02**	3.88E−01	1.18E−01	5.87E−02	2.97E + 00	2.53E + 00	6.58E−02	9.82E−01	6.89E−01	2.54E−02	1.54E + 00
Ave	**2.34E−01**	7.19E−01	4.49E−01	**2.34E−01**	1.69E + 01	2.82E + 00	1.14E + 00	1.66E + 00	1.08E + 00	3.38E + 07	2.15E + 00
Std	1.73E−01	2.16E−01	2.78E−01	1.69E−01	1.17E + 01	**9.70E−02**	5.07E−01	2.83E−01	1.95E−01	1.03E + 08	1.99E−01
W|T| L	**5|4|4**	**0|0|13**	**1|1|11**	**0|0|13**	**1|0|12**	**1|3|9**	**0|2|11**	**0|2|11**	**1|1|11**	**0|0|13**	**0|2|11**

Siginficant values are in bold.

**Table 3:** Results of F14–F23.

	HGWO	GWO	WOA	MVO	SSA	AOA	SOA	GJO	AGWO_CS	PSOGWO	AGWO
F14											
Best	**0.998**	**0.998**	**0.998**	**0.998**	**0.998**	2.982	**0.998**	**0.998**	**0.998**	**0.998**	**0.998**
Ave	3.066	6.036	2.810	**0.998**	1.147	10.072	5.710	4.229	2.967	2.566	5.309
Std	2.80E + 00	4.89E + 00	3.50E + 00	**2.02E−11**	4.86E−01	3.22E + 00	4.54E + 00	4.21E + 00	2.83E + 00	3.26E + 00	4.15E + 00
F15											
Best	**0.0003075**	0.0003078	0.0003404	0.0005344	0.0005216	0.0003433	0.0003435	0.0003076	0.0003095	0.0003100	0.0003078
Ave	**0.0004511**	0.0053934	0.0007942	0.0056669	0.0008628	0.0214637	0.0018627	0.0024570	0.0005651	0.0104350	0.0040555
Std	**1.81E−05**	8.87E−03	5.64E−04	1.34E−02	2.92E−04	3.43E−02	1.59E−03	6.13E−03	3.14E−04	1.02E−02	7.62E−03
F16											
Best	− **1.0316**	− **1.0316**	− **1.0316**	− **1.0316**	− **1.0316**	− **1.0316**	− **1.0316**	− **1.0316**	− **1.0316**	− **1.0316**	− **1.0316**
Ave	− **1.0316**	− **1.0316**	− **1.0316**	− **1.0316**	− **1.0316**	− **1.0316**	− 1.0224	− **1.0316**	− **1.0316**	− **1.0315**	− **1.0316**
Std	**8.82E−17**	1.64E−08	1.02E−09	3.82E−07	4.07E−14	8.45E−08	3.42E−02	3.36E−05	3.16E−06	5.65E−04	1.91E−07
F17											
Best	**0.398**	**0.398**	**0.398**	**0.398**	**0.398**	**0.398**	**0.398**	**0.398**	**0.398**	**0.398**	**0.398**
Ave	**0.398**	**0.398**	**0.398**	**0.398**	**0.398**	0.412	0.406	**0.398**	**0.398**	**0.398**	**0.398**
Std	**2.32E−13**	2.12E−05	1.21E−05	6.81E−07	5.86E−13	1.68E−02	2.40E−02	3.53E−05	6.12E−04	1.46E−04	2.23E−04
F18											
Best	**3.0000**	**3.0000**	**3.0000**	**3.0000**	**3.0000**	**3.0000**	**3.0000**	**3.0000**	**3.0000**	**3.0000**	**3.0000**
Ave	**3.0000**	**3.0000**	3.0001	**3.0000**	**3.0000**	5.7009	11.4133	**3.0000**	**3.0000**	**3.0041**	**3.0000**
Std	**1.07E−14**	4.02E−05	1.29E−04	3.92E−06	2.15E−13	8.31E + 00	1.32E + 01	1.01E−05	1.08E−05	1.81E−02	4.63E−06
F19											
Best	− **3.86**	− **3.86**	− **3.86**	− **3.86**	− **3.86**	− **3.86**	− **3.86**	− **3.86**	− **3.86**	− **3.86**	− **3.86**
Ave	− **3.86**	− **3.86**	− **3.86**	− **3.86**	− **3.86**	− 3.85	− **3.66**	− **3.86**	− **3.86**	− **3.86**	− **3.86**
Std	3.71E−03	1.47E−03	3.83E−03	5.96E−07	**1.78E−11**	5.32E−03	2.67E−01	3.88E−03	6.22E−04	3.36E−03	2.52E−03
F20											
Best	− **3.32**	− **3.32**	− **3.32**	− **3.32**	− **3.32**	− 3.27	− 3.08	− **3.32**	− 3.31	− **3.32**	− **3.32**
Ave	− 3.03	− 3.27	− 3.20	− 3.24	− 3.22	− 3.08	− 2.45	− 3.15	− **3.28**	− 3.16	− 3.27
Std	2.27E−01	7.85E−02	1.39E−01	5.98E−02	5.38E−02	1.05E−01	5.00E−01	1.45E−01	**4.18E−02**	1.39E−01	9.05E−02
F21											
Best	− **10.1532**	− 10.1531	− 10.1511	− 10.1531	− **10.1532**	− 6.9718	− 9.4986	− 10.1494	− 9.4662	− 10.1480	− 10.1460
Ave	− 4.8927	− 7.8998	− 6.9811	− 6.2468	− 6.8941	− 3.8600	− 3.7622	− **8.1266**	− 5.6330	− 7.4352	− 7.3555
Std	**1.18E + 00**	3.13E + 00	3.05E + 00	3.10E + 00	3.45E + 00	1.26E + 00	2.17E + 00	2.88E + 00	1.79E + 00	2.67E + 00	3.27E + 00
F22											
Best	− **10.4029**	− **10.4029**	− 10.4003	− **10.4029**	− **10.4029**	− 9.4367	− 9.8347	− 10.4017	− 9.4603	− 10.4024	− 10.3990
Ave	− **10.1386**	− 9.3326	− 7.0888	− 7.7719	− 8.7252	− 4.4329	− 3.3616	− 9.8616	− 6.4946	− 9.5184	− 8.5985
Std	**1.17E + 00**	1.18E + 00	3.50E + 00	3.42E + 00	3.04E + 00	2.01E + 00	2.01E + 00	1.62E + 00	2.16E + 00	1.96E + 00	3.21E + 00
F23											
Best	− **10.5364**	− 10.5363	− 10.5329	− 10.5363	− **10.5364**	− 7.4739	− 7.4441	− 10.5357	− 9.0298	− 10.5336	− 10.5340
Ave	− 8.1732	− **10.1301**	− 7.6849	− 9.8601	− 7.8288	− 3.8452	− 3.0400	− 9.1755	− 6.9846	− 9.7149	− 9.0480
Std	3.03E + 00	1.81E + 00	3.33E + 00	2.13E + 00	3.80E + 00	**1.58E + 00**	1.72E + 00	2.83E + 00	1.88E + 00	2.10E + 00	3.05E + 00
W|T| L	**6|2|2**	**0|1|9**	**0|1|9**	**1|1|8**	**1|1|8**	**0|0|10**	**0|0|10**	**0|1|9**	**0|0|10**	**0|1|9**	**0|1|9**

Siginficant values are in bold.

#### Comparison with other hybridization algorithms of WGO

**Figure 6:**
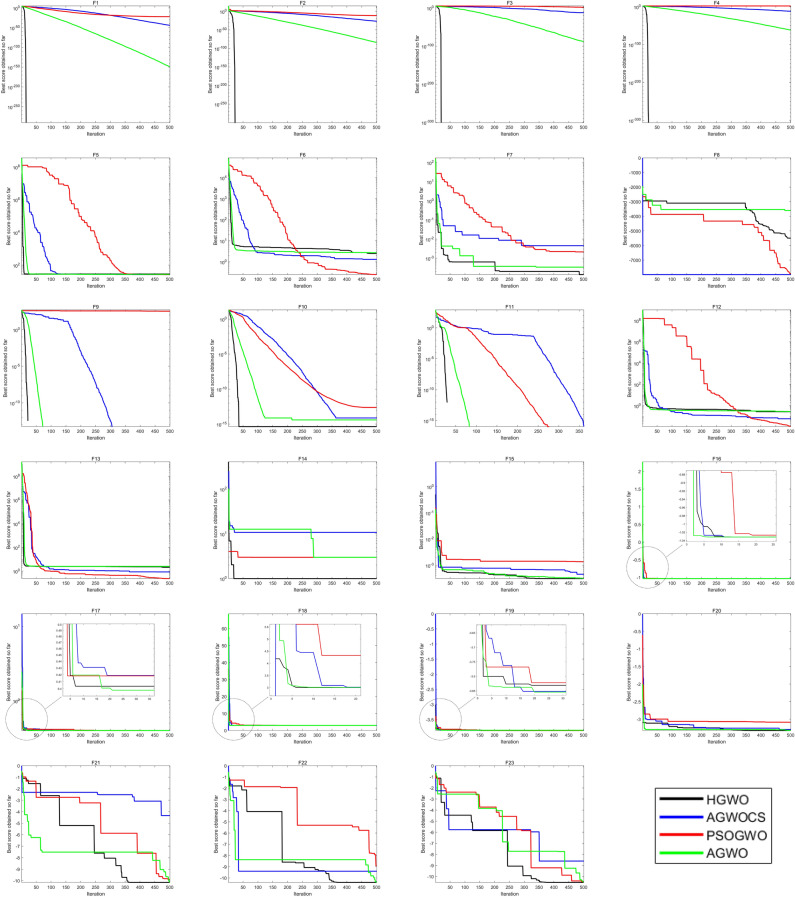
Convergence curve of hybridization algorithm.

To better assess the effectiveness of HGWO, this study compares it with three other hybrid optimization algorithms: AGWO_CS^81^, PSOGWO^82^, and AGWO^63^. The experimental conditions are consistent with those described in the previous paper. The results and convergence curves can be found in Tables 2 and 3, and Fig. 6, respectively.

#### Exploitation analysis

The experimental results of each algorithm on the unimodal benchmark functions are presented in Table 2. Unimodal benchmark functions, which only contain one minimum value, are ideal for testing the exploitation performance of the algorithm^83^. For F1–F4, the HGWO algorithm can achieve the theoretical optimal solution within approximately 50 generations, with a mean and standard deviation of zero. This indicates that the HGWO algorithm demonstrates both strong solution accuracy and stability. The performance results of each algorithm in solving F1–F7 in different dimensions can be found in Tables 4 and 5. Upon comparing the solution outcomes, it becomes evident that the HGWO algorithm proposed in this paper outperforms most of the comparison algorithms.

**Table 4:** Results of F1–F13 (Dim = 50).

	HGWO	GWO	WOA	MVO	SSA	AOA	SOA	GJO	AGWO_CS	PSOGWO	AGWO
F1											
Best	**0.00E + 00**	4.80E−21	1.54E−83	6.23E + 00	6.60E−02	3.43E−44	2.94E−10	4.95E−42	1.43E−32	8.79E−20	1.41E−133
Ave	**0.00E + 00**	7.24E−20	1.48E−71	9.78E + 00	7.09E−01	7.03E−04	2.63E−08	2.92E−40	1.30E−30	1.05E + 04	2.00E−127
Std	**0.00E + 00**	8.50E−20	4.94E−71	1.95E + 00	7.51E−01	1.54E−03	6.25E−08	7.52E−40	2.10E−30	2.74E + 04	5.56E−127
F2											
Best	**0.00E + 00**	8.81E−13	1.12E−58	5.97E + 00	3.76E + 00	6.90E−276	2.94E−07	5.35E−26	1.55E−20	2.19E−11	4.23E−73
Ave	**0.00E + 00**	2.89E−12	1.75E−49	1.45E + 02	8.72E + 00	1.99E−188	1.30E−06	4.94E−25	1.26E−19	3.79E + 14	1.69E−70
Std	**0.00E + 00**	1.94E−12	7.07E−49	1.05E + 02	3.23E + 00	0.00E + 00	9.49E−07	4.51E−25	1.45E−19	1.70E + 15	3.36E−70
F3											
Best	**0.00E + 00**	1.38E−02	1.27E + 05	3.42E + 03	2.20E + 03	1.73E−02	4.97E−04	2.14E−16	3.94E−06	3.99E−03	5.40E−83
Ave	**0.00E + 00**	4.03E−01	1.94E + 05	5.85E + 03	8.47E + 03	8.13E−02	4.64E−01	1.91E−07	1.02E−01	2.44E + 04	1.04E−68
Std	**0.00E + 00**	4.84E−01	3.60E + 04	1.40E + 03	4.02E + 03	5.68E−02	1.16E + 00	5.24E−07	4.23E−01	4.79E + 04	4.67E−68
F4											
Best	**0.00E + 00**	7.17E−05	2.66E + 00	9.92E + 00	1.50E + 01	4.04E−02	7.91E−02	5.36E−11	9.89E−09	1.87E−04	1.21E−57
Ave	**0.00E + 00**	3.76E−04	6.59E + 01	1.61E + 01	2.08E + 01	5.36E−02	8.47E + 00	4.77E−07	2.70E−07	1.65E + 01	2.52E−55
Std	**0.00E + 00**	3.37E−04	2.96E + 01	4.46E + 00	4.14E + 00	9.23E−03	1.66E + 01	1.69E−06	3.81E−07	2.87E + 01	4.63E−55
F5											
Best	4.72E + 01	4.61E + 01	4.75E + 01	2.14E + 02	1.01E + 02	4.81E + 01	4.79E + 01	4.62E + 01	**4.54E + 01**	4.59E + 01	4.72E + 01
Ave	4.84E + 01	4.75E + 01	4.82E + 01	6.22E + 02	2.41E + 03	4.87E + 01	4.85E + 01	4.81E + 01	**4.72E + 01**	2.91E + 07	4.80E + 01
Std	5.19E−01	9.51E−01	3.94E−01	6.53E + 02	5.73E + 03	**2.69E−01**	3.35E−01	7.02E−01	5.72E−01	9.20E + 07	6.55E−01
F6											
Best	6.26E + 00	1.50E + 00	7.69E−01	6.01E + 00	**1.05E−01**	6.18E + 00	5.99E + 00	5.09E + 00	3.54E + 00	2.21E−01	6.69E + 00
Ave	7.43E + 00	2.90E + 00	1.42E + 00	9.27E + 00	**6.45E−01**	7.08E + 00	7.24E + 00	6.13E + 00	4.33E + 00	6.65E + 03	7.44E + 00
Std	**3.44E−01**	7.37E−01	4.80E−01	2.34E + 00	6.13E−01	3.76E−01	6.05E−01	5.10E−01	4.32E−01	2.33E + 04	4.39E−01
F7											
Best	**2.21E−06**	9.42E−04	7.19E−06	4.10E−02	2.29E−01	6.56E−06	5.18E−04	1.13E−04	3.47E−04	1.48E−03	4.70E−05
Ave	**1.55E−04**	3.67E−03	4.33E−03	1.04E−01	5.38E−01	3.94E−04	4.27E−03	8.21E−04	2.51E−03	1.78E + 01	2.94E−04
Std	**1.50E−04**	1.90E−03	3.91E−03	4.55E−02	1.67E−01	3.83E−04	4.65E−03	6.43E−04	1.90E−03	6.63E + 01	2.27E−04
F8											
Best	− 9.12E + 03	− 1.15E + 04	− **2.09E + 04**	− 1.39E + 04	− 1.36E + 04	− 7.61E + 03	− 1.04E + 04	− 8.81E + 03	− 1.35E + 04	− 1.23E + 04	− 5.37E + 03
Ave	− 7.08E + 03	− 8.65E + 03	− **1.68E + 04**	− 1.21E + 04	− 1.17E + 04	− 7.10E + 03	− 6.91E + 03	− 5.95E + 03	− 1.11E + 04	− 1.02E + 04	− 3.97E + 03
Std	1.02E + 03	1.66E + 03	2.97E + 03	9.46E + 02	1.11E + 03	**3.55E + 02**	1.08E + 03	1.87E + 03	1.37E + 03	1.18E + 03	6.05E + 02
F9											
Best	**0.00E + 00**	4.55E−13	**0.00E + 00**	2.08E + 02	5.78E + 01	**0.00E + 00**	8.32E−10	**0.00E + 00**	**0.00E + 00**	3.98E−13	**0.00E + 00**
Ave	**0.00E + 00**	3.19E + 00	**0.00E + 00**	2.74E + 02	8.89E + 01	**0.00E + 00**	6.54E + 00	**0.00E + 00**	2.26E + 00	2.42E + 02	**0.00E + 00**
Std	**0.00E + 00**	5.42E + 00	**0.00E + 00**	4.14E + 01	2.76E + 01	**0.00E + 00**	9.69E + 00	**0.00E + 00**	7.85E + 00	2.97E + 02	**0.00E + 00**
F10											
Best	**4.44E−16**	1.15E−11	**4.44E−16**	2.17E + 00	3.14E + 00	**4.44E−16**	2.00E + 01	7.55E−15	1.47E−14	7.07E−11	4.00E−15
Ave	**4.44E−16**	4.19E−11	3.82E−15	2.97E + 00	4.96E + 00	**4.44E−16**	2.00E + 01	1.09E−14	1.75E−14	4.19E + 00	6.31E−15
Std	**0.00E + 00**	2.28E−11	2.70E−15	3.60E−01	9.45E−01	**0.00E + 00**	1.66E−03	3.15E−15	3.38E−15	7.04E + 00	1.74E−15
F11											
Best	**0.00E + 00**	**0.00E + 00**	**0.00E + 00**	1.06E + 00	1.45E−01	4.31E−01	4.90E−10	**0.00E + 00**	**0.00E + 00**	**0.00E + 00**	**0.00E + 00**
Ave	**0.00E + 00**	5.35E−03	**0.00E + 00**	1.09E + 00	4.25E−01	1.51E + 00	1.34E−02	**0.00E + 00**	1.01E−03	6.68E + 01	**0.00E + 00**
Std	**0.00E + 00**	8.48E−03	**0.00E + 00**	2.06E−02	1.91E−01	1.94E + 00	1.85E−02	**0.00E + 00**	4.51E−03	1.74E + 02	**0.00E + 00**
F12											
Best	4.17E−02	4.94E−02	**7.35E−03**	2.68E + 00	8.02E + 00	6.70E−01	3.90E−01	2.78E−01	1.51E−01	3.83E−02	3.90E−01
Ave	2.60E−01	1.17E−01	**3.17E−02**	6.36E + 00	1.41E + 01	7.20E−01	5.51E−01	4.16E−01	2.18E−01	6.99E + 07	4.90E−01
Std	5.15E−02	4.74E−02	**1.55E−02**	2.33E + 00	4.69E + 00	3.61E−02	1.33E−01	1.18E−01	4.11E−02	1.84E + 08	5.16E−02
F13											
Best	4.08E + 00	1.44E + 00	**6.75E−01**	9.85E−01	5.33E + 01	4.66E + 00	3.25E + 00	3.22E + 00	2.43E + 00	1.00E + 00	3.79E + 00
Ave	4.48E + 00	2.08E + 00	**1.18E + 00**	6.78E + 00	7.64E + 01	4.85E + 00	3.91E + 00	3.57E + 00	2.93E + 00	1.84E + 08	4.14E + 00
Std	1.24E−01	3.40E−01	3.72E−01	7.41E + 00	1.54E + 01	**9.60E−02**	2.74E−01	2.53E−01	2.81E−01	5.46E + 08	1.73E−01
W|T| L	**5|3|5**	**0|0|13**	**3|2|8**	**0|0|13**	**1|0|12**	**0|2|11**	**0|0|13**	**0|2|11**	**1|0|12**	**0|0|13**	**0|2|11**

Siginficant values are in bold.

**Table 5:** Results of F1–F13 (Dim = 100).

	HGWO	GWO	WOA	MVO	SSA	AOA	SOA	GJO	AGWO_CS	PSOGWO	AGWO
F1											
Best	**0.00E + 00**	2.91E−13	5.08E−81	1.01E + 02	8.36E + 02	1.19E−02	1.67E−06	1.60E−29	1.15E−20	2.83E−12	2.07E−112
Ave	**0.00E + 00**	1.49E−12	4.23E−71	1.68E + 02	1.42E + 03	2.51E−02	3.12E−05	2.23E−27	4.36E−19	1.70E + 04	9.82E−108
Std	**0.00E + 00**	1.45E−12	1.77E−70	3.91E + 01	4.25E + 02	7.71E−03	3.06E−05	6.73E−27	6.51E−19	5.23E + 04	2.98E−107
F2											
Best	**0.00E + 00**	2.27E−08	1.49E−56	4.09E + 02	3.53E + 01	1.76E−122	1.49E−05	2.68E−18	1.24E−13	2.56E−07	4.31E−62
Ave	**0.00E + 00**	4.35E−08	1.30E−49	1.31E + 22	4.54E + 01	6.15E−72	6.80E−05	9.76E−18	4.73E−13	1.89E + 35	1.38E−59
Std	**0.00E + 00**	1.34E−08	4.04E−49	4.75E + 22	7.00E + 00	2.43E−71	4.72E−05	6.33E−18	2.95E−13	7.39E + 35	2.63E−59
F3											
Best	**0.00E + 00**	3.48E + 01	5.06E + 05	4.54E + 04	1.79E + 04	3.09E−01	2.83E−01	1.16E−06	1.00E−01	2.32E + 02	6.91E−70
Ave	**0.00E + 00**	4.59E + 02	1.08E + 06	6.76E + 04	5.45E + 04	7.63E−01	1.73E + 02	2.70E + 00	1.98E + 03	1.23E + 05	3.69E−45
Std	**0.00E + 00**	5.77E + 02	3.17E + 05	1.23E + 04	3.36E + 04	5.47E−01	3.33E + 02	1.07E + 01	2.74E + 03	1.90E + 05	1.65E−44
F4											
Best	**0.00E + 00**	2.30E−01	1.43E−01	4.98E + 01	2.27E + 01	8.23E−02	5.50E + 01	9.58E−04	5.17E−04	2.30E−02	4.21E−51
Ave	**0.00E + 00**	1.34E + 00	7.49E + 01	5.73E + 01	2.77E + 01	9.30E−02	7.88E + 01	4.84E + 00	5.72E−01	2.55E + 01	7.28E−50
Std	**0.00E + 00**	1.11E + 00	3.10E + 01	4.72E + 00	4.82E + 00	9.49E−03	1.03E + 01	6.37E + 00	2.37E + 00	3.71E + 01	1.12E−49
F5											
Best	9.72E + 01	**9.60E + 01**	9.74E + 01	3.29E + 03	6.46E + 04	9.87E + 01	9.80E + 01	9.71E + 01	9.71E + 01	9.65E + 01	9.72E + 01
Ave	9.85E + 01	**9.78E + 01**	9.82E + 01	1.34E + 04	1.63E + 05	9.89E + 01	9.85E + 01	9.83E + 01	9.79E + 01	4.80E + 07	9.84E + 01
Std	4.25E−01	7.13E−01	2.78E−01	1.17E + 04	9.16E + 04	**6.33E**−**02**	3.13E−01	5.43E−01	4.64E−01	1.60E + 08	4.89E−01
F6											
Best	1.75E + 01	8.94E + 00	**1.80E + 00**	1.29E + 02	7.52E + 02	1.71E + 01	1.72E + 01	1.47E + 01	1.26E + 01	6.14E + 00	1.88E + 01
Ave	1.91E + 01	1.03E + 01	**3.66E + 00**	1.67E + 02	1.43E + 03	1.84E + 01	1.85E + 01	1.62E + 01	1.45E + 01	2.06E + 04	1.95E + 01
Std	7.90E−01	8.38E−01	1.11E + 00	2.62E + 01	4.00E + 02	4.74E−01	6.06E−01	1.04E + 00	6.88E−01	5.25E + 04	**3.76E**−**01**
F7											
Best	**2.16E**−**06**	2.59E−03	8.03E−05	4.54E−01	1.38E + 00	6.38E−06	3.62E−03	2.00E−04	6.15E−04	5.08E−03	2.04E−05
Ave	**5.61E**−**05**	5.61E−03	6.28E−03	6.38E−01	2.87E + 00	1.24E−−04	1.09E−02	1.41E−03	3.86E−03	8.84E + 01	3.51E−04
Std	**3.64E**−**05**	1.96E−03	7.11E−03	1.12E−01	6.98E−01	1.13E−04	7.26E−03	8.92E−04	2.18E−03	2.96E + 02	2.39E−04
F8											
Best	−1.15E + 04	−1.85E + 04	−**4.18E + 04**	−2.64E + 04	−2.50E + 04	−1.16E + 04	−1.40E + 04	−1.63E + 04	−2.54E + 04	−2.00E + 04	−8.49E + 03
Ave	−7.10E + 03	−1.54E + 04	−**3.53E + 04**	−2.34E + 04	−2.17E + 04	−1.02E + 04	−9.88E + 03	−1.02E + 04	−2.29E + 04	−1.67E + 04	−5.86E + 03
Std	1.89E + 03	2.53E + 03	6.10E + 03	1.34E + 03	1.64E + 03	**7.81E + 02**	1.43E + 03	3.99E + 03	1.67E + 03	1.44E + 03	8.57E + 02
F9											
Best	**0.00E + 00**	5.03E−09	**0.00E + 00**	6.37E + 02	1.75E + 02	**0.00E + 00**	2.72E−07	**0.00E + 00**	**0.00E + 00**	4.66E−09	**0.00E + 00**
Ave	**0.00E + 00**	9.95E + 00	**0.00E + 00**	7.17E + 02	2.38E + 02	**0.00E + 00**	3.49E + 00	**0.00E + 00**	5.10E−01	4.42E + 02	**0.00E + 00**
Std	**0.00E + 00**	7.18E + 00	**0.00E + 00**	7.98E + 01	4.33E + 01	**0.00E + 00**	4.08E + 00	**0.00E + 00**	2.28E + 00	5.67E + 02	**0.00E + 00**
F10											
Best	**4.44E**−**16**	8.36E−08	**4.44E**−**16**	4.34E + 00	7.96E + 00	**4.44E**−**16**	2.00E + 01	3.95E−14	1.61E−11	1.64E−07	4.00E−15
Ave	**4.44E**−**16**	1.35E−07	4.35E−15	9.53E + 00	9.88E + 00	1.69E−04	2.00E + 01	4.79E−14	7.98E−11	3.48E + 00	7.02E−15
Std	**0.00E + 00**	3.17E−08	3.03E−15	7.11E + 00	1.25E + 00	6.31E−04	3.60E−04	8.25E−15	8.04E−11	7.48E + 00	1.30E−15
F11											
Best	**0.00E + 00**	1.05E−13	**0.00E + 00**	2.05E + 00	9.54E + 00	3.09E + 02	5.27E−07	**0.00E + 00**	**0.00E + 00**	1.75E−12	**0.00E + 00**
Ave	**0.00E + 00**	5.38E−03	**0.00E + 00**	2.46E + 00	1.32E + 01	6.15E + 02	1.36E−02	**0.00E + 00**	1.86E−03	8.76E + 01	**0.00E + 00**
Std	**0.00E + 00**	9.75E−03	**0.00E + 00**	2.32E−01	2.81E + 00	1.76E + 02	2.84E−02	**0.00E + 00**	8.32E−03	2.59E + 02	**0.00E + 00**
F12											
Best	7.84E−01	2.49E−01	**2.00E**−**02**	1.33E + 01	1.50E + 01	8.73E−01	6.43E−01	4.85E−01	3.76E−01	1.26E−01	5.98E−01
Ave	9.53E−01	3.25E−01	**5.54E**−**02**	2.22E + 01	3.36E + 01	9.07E−01	7.82E−01	6.05E−01	4.90E−01	1.13E + 08	7.34E−01
Std	7.97E−02	7.70E−02	4.09E−02	6.84E + 00	1.25E + 01	**2.25E**−**02**	7.22E−02	5.93E−02	6.42E−02	4.18E + 08	4.07E−02
F13											
Best	9.28E + 00	6.02E + 00	**1.53E + 00**	1.19E + 02	2.85E + 02	9.84E + 00	9.08E + 00	7.73E + 00	7.32E + 00	5.72E + 00	9.01E + 00
Ave	9.55E + 00	6.82E + 00	**2.96E + 00**	1.66E + 02	2.54E + 03	9.96E + 00	9.47E + 00	8.35E + 00	7.97E + 00	7.61E + 08	9.30E + 00
Std	1.33E−01	5.11E−01	1.12E + 00	1.97E + 01	2.09E + 03	**5.71E**−**02**	3.08E−01	2.53E−01	2.59E−01	1.57E + 09	1.47E−01
W|T| L	**6|2|5**	**1|0|12**	**4|2|7**	**0|0|13**	**0|0|13**	**0|1|12**	**0|0|13**	**0|2|11**	**0|0|13**	**0|0|13**	**0|2|11**

Siginficant values are in bold.

#### Exploration analysis

The benchmark functions F8–F13 are multimodal and exhibit multiple extremes that increase with dimensionality. They serve as effective tools for evaluating an algorithm's exploration ability and its capacity to escape local optima^83^. Tables 2, 4, and 5 compare each algorithm's performance in solving F8–F13 in various dimensions. Despite the increasing difficulty with higher dimensionality, the HGWO algorithm consistently performs exceptionally well in most of the multi-peaked functions, showing no major fluctuations in stability. The multi-peaked function is known to have numerous local optimal solutions. The favorable experimental results presented in the table provide quantitative evidence of the HGWO algorithm's ability to effectively escape these local optima during the optimization process.

The F14–F23 benchmark functions are fixed-dimension multimodal problems. Table 3 demonstrates that the majority of algorithms are capable of locating the theoretical optimum. However, the HGWO algorithm outperforms most algorithms in terms of mean and standard deviation, indicating strong and stable search performance.

The convergence curves of various algorithms for solving the benchmark functions are depicted in Figs. 5 and 6. It is evident that the HGWO algorithm outperforms the majority of algorithms in terms of convergence speed for both unimodal and multimodal functions. Additionally, the HGWO algorithm possesses the capability to escape local optima by utilizing its variable position update formula, particularly in F1–F4 and F9–F11, where it can achieve the theoretical optimum or a value close to it within approximately 50 iterations.

#### Statistical analysis

In the previous statistical process, we determined the optimal value, mean, and standard deviation of 20 experimental results for each algorithm. This allowed us to compare the superiority of the algorithms. To further compare the differences between the HGWO and other algorithms, we conducted a statistical analysis using the Wilcoxon rank-sum test with a significance level of 5%, based on the aforementioned simulations. The resulting *P*-values from the experiments are presented in Table 6.

**Table 6:** *P*-values for Wilcoxon rank sum test.

	GWO	WOA	MVO	SSA	AOA	SOA	GJO	AGWO_CS	PSOGWO	AGWO
F1	8.01E−09	8.01E−09	8.01E−09	8.01E−09	8.01E−09	8.01E−09	8.01E−09	8.01E−09	8.01E−09	8.01E−09
F2	8.01E−09	8.01E−09	8.01E−09	8.01E−09	**NaN**	8.01E−09	8.01E−09	8.01E−09	8.01E−09	8.01E−09
F3	8.01E−09	8.01E−09	8.01E−09	8.01E−09	8.01E−09	8.01E−09	8.01E−09	8.01E−09	8.01E−09	8.01E−09
F4	8.01E−09	8.01E−09	8.01E−09	8.01E−09	8.01E−09	8.01E−09	8.01E−09	8.01E−09	8.01E−09	8.01E−09
F5	3.07E−06	**9.46E**−**01**	6.80E−08	1.04E−04	2.94E−02	8.10E−02	**9.03E**−**01**	5.25E−05	3.65E−01	**7.76E**−**01**
F6	6.80E−08	6.80E−08	6.80E−08	6.80E−08	**7.56E**−**01**	**5.43E**−**01**	2.23E−02	6.80E−08	3.15E−02	**6.75E**−**01**
F7	6.80E−08	1.92E−07	6.80E−08	6.80E−08	3.85E−02	6.80E−08	5.25E−05	6.80E−08	6.80E−08	3.65E−01
F8	**6.95E**−**01**	7.90E−08	2.96E−07	1.38E−06	8.36E−04	1.78E−03	6.01E−07	1.16E−04	4.32E−03	6.80E−08
F9	7.79E−09	3.42E−01	8.01E−09	8.01E−09	**NaN**	8.01E−09	**NaN**	3.42E−01	7.99E−09	**NaN**
F10	7.59E−09	5.74E−05	8.01E−09	8.01E−09	**NaN**	8.01E−09	3.10E−09	7.43E−10	8.01E−09	7.43E−10
F11	1.98E−02	3.42E−01	8.01E−09	8.01E−09	8.01E−09	8.01E−09	**NaN**	3.42E−01	1.05E−07	**NaN**
F12	6.80E−08	6.80E−08	3.99E−06	6.80E−08	3.07E−06	**8.18E**−**01**	6.67E−06	6.80E−08	1.20E−01	6.04E−03
F13	6.80E−08	6.80E−08	6.80E−08	9.21E−04	3.42E−07	3.38E−04	1.66E−07	6.80E−08	7.11E−03	1.55E−02
F14	3.65E−01	3.94E−01	7.08E−03	1.04E−03	1.40E−05	**9.68E**−**01**	4.97E−02	1.48E−01	5.64E−02	8.08E−02
F15	1.14E−01	2.34E−03	3.07E−06	1.38E−06	1.16E−04	6.80E−08	5.56E−03	3.06E−03	1.06E−02	4.41E−01
F16	3.96E−08	3.96E−08	3.96E−08	3.95E−08	3.96E−08	3.96E−08	3.96E−08	3.96E−08	3.96E−08	3.96E−08
F17	1.13E−08	1.13E−08	1.13E−08	2.68E−06	1.13E−08	1.13E−08	1.13E−08	1.13E−08	1.13E−08	1.13E−08
F18	6.72E−08	6.72E−08	6.72E−08	6.72E−08	6.72E−08	6.72E−08	6.72E−08	6.72E−08	6.72E−08	6.72E−08
F19	6.35E−03	**7.94E**−**01**	6.35E−03	6.09E−03	1.30E−04	3.34E−06	3.44E−01	6.35E−03	**7.94E**−**01**	5.66E−02
F20	2.47E−04	3.64E−03	1.61E−04	4.68E−05	1.20E−01	2.50E−01	7.20E−02	3.05E−04	1.20E−01	1.48E−03
F21	8.36E−04	4.57E−01	4.57E−01	1.33E−02	9.75E−06	9.05E−03	4.11E−02	**5.98E**−**01**	1.08E−01	4.11E−02
F22	7.11E−03	4.57E−01	1.33E−01	9.78E−03	8.59E−06	3.60E−02	6.01E−02	2.62E−01	2.23E−02	1.40E−01
F23	2.08E−01	1.90E−01	**7.76E**−**01**	2.50E−01	5.63E−04	1.33E−01	1.99E−01	**6.36E**−**01**	1.72E−01	**5.79E**−**01**

Siginficant values are in bold.

The Wilcoxon rank sum test is a non-parametric statistical test that determines the rejection of the null hypothesis and indicates the significance of HGWO in comparison to other algorithms. If the *P*-value is less than 0.05, it can be considered strong evidence that HGWO is more significant than other algorithms. Conversely, if the *P*-value is greater than 0.05, it indicates that HGWO is less significant than other algorithms (indicated in bold in the table). When the *P*-value is exactly 0.05, the result is noted as "NaN" indicating that a significance judgement could not be made. As the algorithms cannot be compared to themselves, the table no longer includes the *P*-values of HGWO.

The findings presented in Table 6 demonstrate that the *P*-values of the test conducted between HGWO and the comparison algorithms, for all 23 benchmark functions, are less than 0.05 in over 95% of cases. As a result, the null hypothesis is rejected, indicating a significant difference between the computation outcomes of HGWO and the other 10 algorithms.

### Compare results using CEC2020

To further investigate the quality of the HGWO and assess its ability for exploration, exploitation, and avoidance of local optima, we employed the CEC2020 suite, which is renowned for its challenging benchmarks. This suite encompasses four types of functions: unimodal function (F24), basic functions (F25–F27), hybrid functions (F28–F30), and composition functions (F31–F33). We evaluated the performance of HGWO using these benchmarks and compared it with well-known algorithms such as GWO, WOA, SSA, and AGWO. Each algorithm underwent 20 independent runs with 500 iterations and 30 search agents.

As presented in Table 7, our findings indicate that HGWO outperforms other algorithms in 5 test functions and demonstrates comparable performance to excellent heuristic algorithms in 5 test functions. Consequently, we conclude that HGWO can be classified as a superior optimizer.

**Table 7:** Results of CEC2020.

	HGWO	GWO	WOA	MVO	SSA	AOA	SOA	GJO	AGWO_CS	PSOGWO	AGWO
F24											
Best	5.14E + 03	7.45E + 03	9.11E + 06	5.95E + 03	**1.02E + 02**	2.21E + 09	1.71E + 07	3.31E + 06	2.21E + 07	2.55E + 05	1.79E + 07
Ave	1.06E + 07	1.09E + 08	5.42E + 07	1.86E + 04	**1.90E + 03**	7.59E + 09	4.44E + 08	4.57E + 08	2.13E + 08	1.22E + 08	3.30E + 08
Std	1.34E + 07	1.69E + 08	6.34E + 07	9.23E + 03	**2.17E + 03**	3.95E + 09	2.56E + 08	3.56E + 08	1.71E + 08	2.76E + 08	3.87E + 08
F25											
Best	**1.13E + 03**	1.31E + 03	1.60E + 03	1.44E + 03	1.27E + 03	1.85E + 03	1.65E + 03	1.73E + 03	1.51E + 03	1.24E + 03	1.78E + 03
Ave	**1.31E + 03**	1.59E + 03	2.24E + 03	1.82E + 03	2.00E + 03	2.33E + 03	2.02E + 03	2.17E + 03	2.10E + 03	1.81E + 03	2.08E + 03
Std	**1.54E + 02**	1.91E + 02	3.33E + 02	2.62E + 02	2.75E + 02	2.47E + 02	2.51E + 02	4.02E + 02	2.89E + 02	3.72E + 02	2.06E + 02
F26											
Best	**7.12E + 02**	7.14E + 02	7.46E + 02	7.16E + 02	7.14E + 02	7.52E + 02	7.43E + 02	7.26E + 02	7.44E + 02	7.15E + 02	7.44E + 02
Ave	**7.31E + 02**	7.35E + 02	7.98E + 02	**7.31E + 02**	7.39E + 02	7.97E + 02	7.73E + 02	7.55E + 02	7.56E + 02	7.48E + 02	7.63E + 02
Std	**1.07E + 00**	1.34E + 01	3.26E + 01	1.12E + 01	1.57E + 01	1.57E + 01	1.78E + 01	2.04E + 01	8.67E + 00	4.21E + 01	1.24E + 01
F27											
Best	**1.90E + 03**	**1.90E + 03**	**1.90E + 03**	**1.90E + 03**	**1.90E + 03**	7.77E + 03	**1.90E + 03**	**1.90E + 03**	1.91E + 03	**1.90E + 03**	**1.90E + 03**
Ave	**1.90E + 03**	**1.90E + 03**	1.91E + 03	**1.90E + 03**	**1.90E + 03**	2.23E + 05	1.91E + 03	2.20E + 03	2.10E + 03	1.91E + 03	2.11E + 03
Std	**2.46E**−**01**	6.17E + 00	4.58E + 00	7.01E−01	8.85E−01	1.35E + 05	2.58E + 00	7.47E + 02	8.20E + 02	3.62E + 01	7.69E + 02
F28											
Best	**2.30E + 03**	2.57E + 03	1.01E + 04	2.99E + 03	3.08E + 03	3.32E + 04	4.13E + 03	3.13E + 03	5.00E + 03	2.68E + 03	3.07E + 03
Ave	2.43E + 04	6.57E + 04	3.52E + 05	**7.01E + 03**	3.35E + 04	3.73E + 05	1.14E + 05	5.51E + 04	4.71E + 04	6.02E + 04	2.59E + 05
Std	2.27E + 04	1.47E + 05	5.52E + 05	**3.65E + 03**	4.71E + 04	1.78E + 05	1.75E + 05	1.44E + 05	1.08E + 05	1.42E + 05	2.61E + 05
F29											
Best	**1.60E + 03**	**1.60E + 03**	**1.60E + 03**	**1.60E + 03**	**1.60E + 03**	**1.60E + 03**	**1.60E + 03**	**1.60E + 03**	**1.60E + 03**	**1.60E + 03**	**1.60E + 03**
Ave	**1.60E + 03**	1.61E + 03	1.61E + 03	1.61E + 03	**1.60E + 03**	1.61E + 03	**1.60E + 03**	1.61E + 03	1.61E + 03	1.61E + 03	1.61E + 03
Std	**2.69E**−**01**	7.68E + 00	1.35E + 01	7.46E + 00	5.46E + 00	1.09E + 01	3.61E−01	1.40E + 01	1.40E + 01	1.37E + 01	9.36E + 00
F30											
Best	**2.28E + 03**	2.73E + 03	1.34E + 04	2.28E + 03	2.45E + 03	3.86E + 03	3.44E + 03	3.10E + 03	4.70E + 03	3.37E + 03	3.10E + 03
Ave	8.05E + 03	8.92E + 03	6.04E + 05	8.66E + 03	6.70E + 03	1.03E + 06	1.13E + 04	9.62E + 03	1.12E + 04	1.44E + 04	4.53E + 04
Std	**3.53E + 03**	4.99E + 03	1.20E + 06	6.96E + 03	5.09E + 03	2.07E + 06	9.13E + 03	6.73E + 03	3.96E + 03	8.26E + 03	7.24E + 04
F31											
Best	2.30E + 03	2.30E + 03	**2.20E + 03**	2.23E + 03	2.23E + 03	2.65E + 03	2.23E + 03	2.31E + 03	2.25E + 03	2.30E + 03	2.31E + 03
Ave	2.31E + 03	2.31E + 03	2.38E + 03	**2.30E + 03**	**2.30E + 03**	2.99E + 03	2.89E + 03	2.38E + 03	2.32E + 03	2.35E + 03	2.35E + 03
Std	**8.18E + 00**	1.34E + 01	2.86E + 02	1.72E + 01	1.68E + 01	2.44E + 02	6.52E + 02	9.78E + 01	2.15E + 01	1.00E + 02	4.00E + 01
F32											
Best	2.74E + 03	2.55E + 03	2.57E + 03	2.73E + 03	**2.50E + 03**	2.77E + 03	2.74E + 03	2.56E + 03	2.52E + 03	2.74E + 03	2.74E + 03
Ave	2.75E + 03	2.74E + 03	2.75E + 03	2.75E + 03	**2.74E + 03**	2.87E + 03	2.76E + 03	2.76E + 03	2.70E + 03	2.76E + 03	2.79E + 03
Std	**1.05E + 01**	6.81E + 01	8.36E + 01	1.11E + 01	5.77E + 01	4.80E + 01	1.30E + 01	5.13E + 01	1.13E + 02	1.50E + 01	2.35E + 01
F33											
Best	**2.90E + 03**	**2.90E + 03**	2.92E + 03	**2.90E + 03**	**2.90E + 03**	3.22E + 03	2.91E + 03	2.91E + 03	2.92E + 03	**2.90E + 03**	2.91E + 03
Ave	**2.91E + 03**	2.94E + 03	2.97E + 03	2.92E + 03	2.93E + 03	3.43E + 03	2.95E + 03	2.96E + 03	2.94E + 03	2.94E + 03	2.95E + 03
Std	**1.14E + 01**	1.82E + 01	4.35E + 01	2.41E + 01	2.37E + 01	1.84E + 02	3.58E + 01	4.97E + 01	1.40E + 01	3.26E + 01	3.08E + 01
W|T| L	**5|5|0**	**0|1|9**	**0|0|10**	**0|1|9**	**2|2|6**	**0|0|10**	**0|1|9**	**0|0|10**	**0|0|10**	**0|0|10**	**0|0|10**

Siginficant values are in bold.

### Impact analysis of the modifications

Experimental and statistical results have thus far demonstrated that the Hunting and Gathering Optimization algorithm (HGWO) surpasses other comparative algorithms. In this subsection, we will analyze the impact of several factors on HGWO's performance, including the Latin Hypercube Sampling (LHS) method used for population initialization, the convergence factor of local perturbations, and the enhancement of the position update formula. To assess these factors, we integrate improvement strategies into the GWO algorithm separately, denoting them as GWO1, GWO2, and GWO3, respectively. The results of solving the CEC2020 test function with each individual improvement strategy are presented in Table 8. Importantly, the fundamental parameter settings remain unchanged from the previous section.

**Table 8:** Impact analysis of the modifications.

	GWO	GWO1	GWO2	GWO3	HGWO
F24					
Best	7.45E + 03	7.45E + 03	5.04E + 03	1.69E + 03	5.14E + 03
Ave	1.09E + 08	7.89E + 07	2.27E + 07	2.54E + 07	1.06E + 07
Std	1.69E + 08	1.97E + 08	8.00E + 07	7.53E + 07	1.34E + 07
F25					
Best	1.31E + 03	1.31E + 03	1.26E + 03	1.14E + 03	1.13E + 03
Ave	1.59E + 03	1.66E + 03	1.67E + 03	1.72E + 03	1.31E + 03
Std	1.91E + 02	2.00E + 02	2.76E + 02	2.49E + 02	1.54E + 02
F26					
Best	7.14E + 02	7.12E + 02	7.13E + 02	7.10E + 02	7.12E + 02
Ave	7.35E + 02	7.32E + 02	7.40E + 02	7.36E + 02	7.31E + 02
Std	1.34E + 01	1.19E + 01	1.33E + 01	1.00E + 01	1.07E + 00
F27					
Best	1.90E + 03	1.90E + 03	1.90E + 03	1.90E + 03	1.90E + 03
Ave	1.90E + 03	1.90E + 03	1.90E + 03	1.91E + 03	1.90E + 03
Std	6.17E + 00	1.17E + 00	2.23E + 00	2.52E + 01	2.46E−01
F28					
Best	2.57E + 03	2.55E + 03	2.22E + 03	2.51E + 03	2.30E + 03
Ave	6.57E + 04	1.10E + 04	1.13E + 05	1.98E + 04	2.43E + 04
Std	1.47E + 05	1.86E + 05	1.70E + 05	2.69E + 04	2.27E + 04
F29					
Best	1.60E + 03	1.60E + 03	1.60E + 03	1.60E + 03	1.60E + 03
Ave	1.61E + 03	1.61E + 03	1.61E + 03	1.60E + 03	1.60E + 03
Std	7.68E + 00	7.32E + 00	9.09E + 00	3.39E−01	2.69E−01
F30					
Best	2.73E + 03	2.56E + 03	2.33E + 03	2.46E + 03	2.28E + 03
Ave	8.92E + 03	6.23E + 03	1.07E + 04	7.28E + 03	8.05E + 03
Std	4.99E + 03	4.67E + 03	7.54E + 03	3.35E + 03	3.53E + 03
F31					
Best	2.30E + 03	2.30E + 03	2.22E + 03	2.30E + 03	2.30E + 03
Ave	2.31E + 03	2.31E + 03	2.31E + 03	2.31E + 03	2.31E + 03
Std	1.34E + 01	8.91E + 00	1.99E + 01	9.59E + 00	8.18E + 00
F32					
Best	2.55E + 03	2.53E + 03	2.73E + 03	2.55E + 03	2.74E + 03
Ave	2.74E + 03	2.74E + 03	2.76E + 03	2.74E + 03	2.75E + 03
Std	6.81E + 01	1.28E + 01	1.71E + 01	4.75E + 01	1.05E + 01
F33					
Best	2.90E + 03	2.90E + 03	2.90E + 03	2.90E + 03	2.90E + 03
Ave	2.94E + 03	2.93E + 03	2.94E + 03	2.93E + 03	2.91E + 03
Std	1.82E + 01	1.70E + 01	1.32E + 01	0.07E + 01	1.14E + 01

In the CEC2020 test functions, significant improvements in performance are achieved when individual improvement strategies are added to GWO1, GWO2, and GWO3. Specifically, the LHS method has a limited effect on improving GWO1, as it only enhances the uniformity of the population distribution at the start of the iteration and does not contribute to subsequent iterations. However, GWO2 demonstrates good performance improvements on nine functions, with the exception of the optimal solution and the mean value of F9, where it does not perform better. Overall, the results in Table 8 show satisfactory outcomes, particularly for GWO3. The improved position update formulas in GWO3 effectively enhance the algorithm's convergence accuracy and speed.

### Ethical approval

Written informed consent for publication of this paper was obtained from the Shenyang University and all authors.

## Real application of HGWO in engineering

To assess the applicability of HGWO in real-world optimization problems, this section employs HGWO to tackle various challenges associated with the design of welded beams, pressure vessels, double bar trusses, and reducers. These engineering optimization problems exhibit varying levels of solution difficulties and complex constraints. By comparing the solution outcomes of the HGWO with those of the 10 algorithms discussed in the prior paper, the effectiveness and applicability of the HGWO in solving engineering design optimization problems can be properly evaluated. The mathematical description of these engineering optimization problems is provided in the Online Appendix.

### Constraint optimization problems

Constraint optimization problems are a common class of mathematical planning problems in engineering applications. They are of practical importance due to their difficulty in solving and their wide range of applications in various fields. The constrained optimization problem can be represented by a unified mathematical equation.

21 $$ \begin{array}{*{20}c} {\;\;\;\;\;\;\min f({\varvec{x}})} \\ {{\text{s}}{\text{.t}}{.}\quad \quad g_{i} ({\varvec{x}}) \le 0,i = 1,2, \cdots ,p} \\ {\;\;\;\;\;\;\;h_{j} ({\varvec{x}}) = 0,j = 1,2, \cdots ,q} \\ {{\varvec{x}} \in X} \\ \end{array} $$

where $X$ is the search space of the constrained optimization problem, $g_{i} ({\varvec{x}})$ represents the inequality constraint, and $h_{j} ({\varvec{x}})$ represents the equation constraint.

This paper examines the challenge of solving constrained optimization problems by introducing the FAD (feasibility and dominance) criterion. The FAD criterion acts as a mechanism for handling constraints, allowing for the effective resolution of the complexities associated with them^84^. When assessing the performance of search agents, the constraint violation value (CV) is added exclusively to the original fitness function value. The formula for an infeasible solution is presented below.

22 $$ CV(X) = \sum\limits_{i = 1}^{p} {\max \left( {0,g_{i} \left( X \right)} \right) + \sum\limits_{j = 1}^{q} {\left( {h_{i} \left( X \right)} \right)} } $$

23 $$ \left\{ \begin{gathered} f\left( {X_{1} } \right) < f\left( {x_{2} } \right)\;\;\;\;\;\;\;\;\;\;\;CV\left( {X_{1} } \right) = CV\left( {X_{2} } \right) = 0 \hfill \\ CV\left( {X_{1} } \right) = 0\;\;\;\;\;\;\;\;\;\;\;\;\;\;\;\;CV\left( {X_{2} } \right) \ne 0 \hfill \\ CV\left( {X_{1} } \right) < CV\left( {x_{2} } \right)\;\;\;\;\;\;CV\left( {X_{1} } \right) \ne 0,CV\left( {X_{1} } \right) \ne 0 \hfill \\ \end{gathered} \right. $$

In the search agent iteration, the appropriate individual is selected using Eq. (23) to achieve a feasible solution with a CV value of zero. If both individuals are not feasible solutions, the individual with the smaller CV value is chosen.

### Pressure vessel design

**Figure 7:**
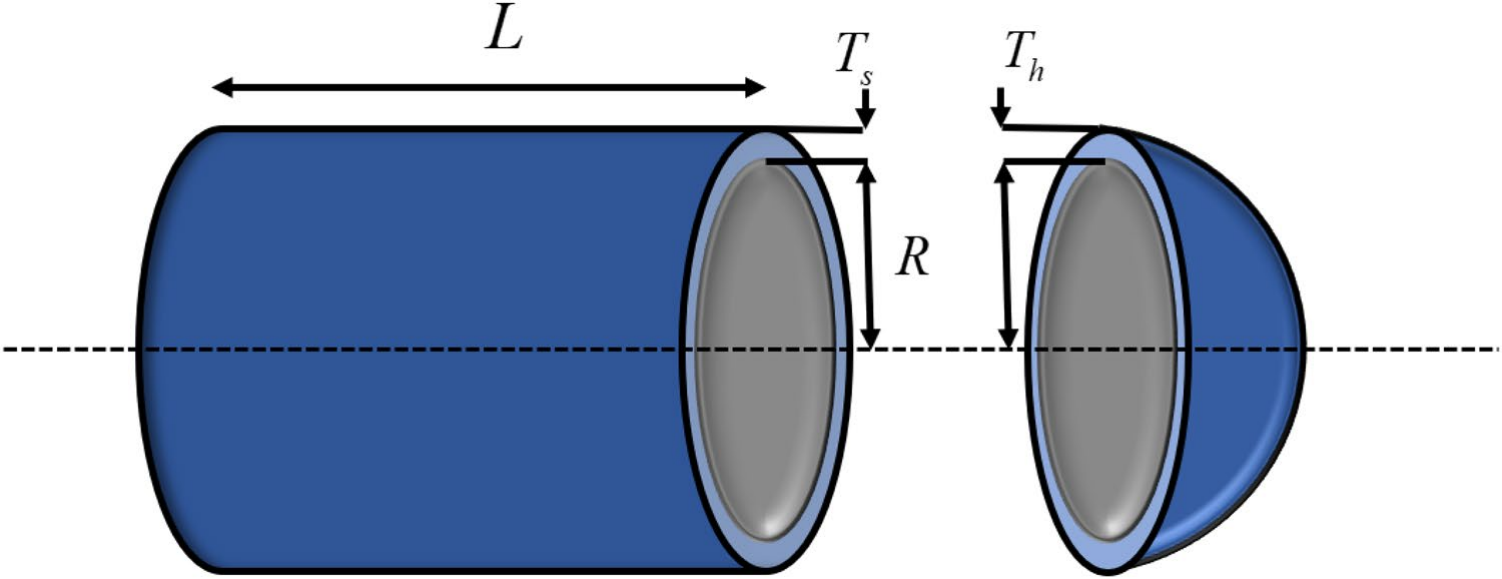
Pressure Vessel Design.

The structure of the pressure vessel design (PVD) is shown in Fig. 7, and its goal is to meet the production needs while minimizing the total cost, with four design variables: shell thickness $T_{s} (x_{{1}} )$, head thickness $T_{h} (x_{{2}} )$, inner radius $R(x_{{3}} )$ and vessel length $L(x_{{4}} )$, where $T_{s} (x_{{1}} )$ and $T_{h} (x_{{2}} )$ are integer multiples of 0.625, and $R(x_{{3}} )$ and $L(x_{{4}} )$ are continuous variables^85^.

Table 9 shows the minimum cost and optimal solution for each pressure vessel design algorithm used. It is clear that HGWO outperforms the other methods in solving pressure vessel design problems with the lowest total cost.

**Table 9:** Experimental results on PVD.

Algorithm	$R(x_{1} )$	$L(x_{2} )$	$T_{s} (x_{3} )$	$T_{h} (x_{4} )$	Cost vaule
HGWO	0.778168733	0.384649205	40.3196231	200	**5885.334276**
GWO	0.779688403	0.387785943	40.38649328	199.1611131	5898.383655
WOA	1.148437469	0.578359633	58.02964415	45.13256509	7041.993924
MVO	0.858675942	0.431426047	44.38555322	152.7915004	6141.690794
SSA	0.796361873	0.393646371	41.26225239	188.6157542	5947.149858
AOA	1.711802352	0.616549339	47.19981739	153.1479006	14308.68337
SOA	1.074290345	0.787991907	55.64704348	59.16433073	8030.451792
GJO	0.784357353	0.396271545	40.52268031	197.5615109	5944.730715
AGWO_CS	0.867251068	0.428920161	44.46543992	151.0756223	6157.224962
PSOGWO	0.782037861	0.391579365	40.50616777	197.8381806	5917.550142
AGWO	0.785142661	0.38746736	40.48970852	198.2045129	5933.249988

Siginficant values are in bold.

### Welded beam design

**Figure 8:**
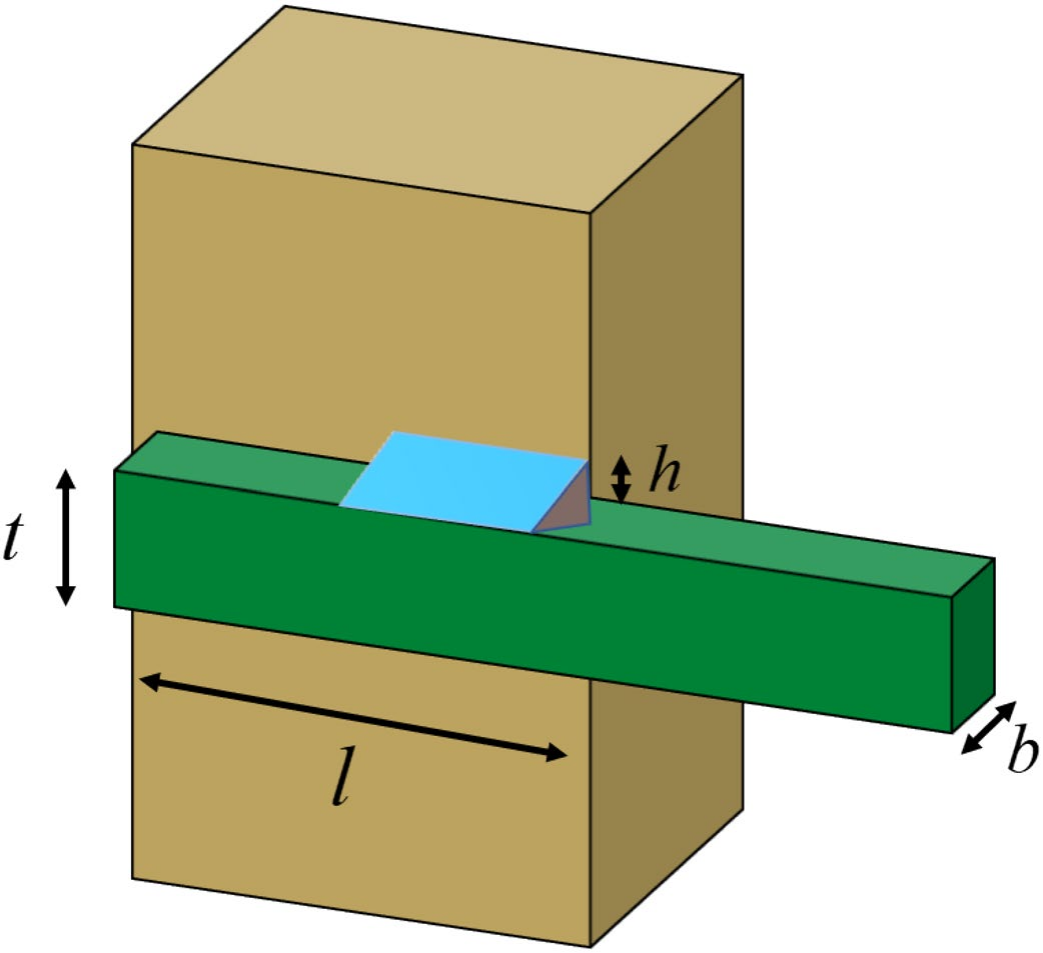
Welded Beam Design.

The design of the welded beam (WBD) is presented in Fig. 8. The optimization problem aims to identify four design variables that meet the restrictions of tangential stress $\tau$, bending stress $\theta$, beam bending load $P_{c}$, end deviation $\delta$, and boundary conditions. These variables include the beam's length $l(x_{2} )$, height $t(x_{3} )$, thickness $b(x_{4} )$, and weld thickness $h(x_{1} )$. The objective is to minimize production costs of the welded beam and the issue is a standard nonlinear programming problem^86^.

Examining Table 10 reveals that the majority of algorithms yield enhanced outcomes for the welded beam design problem. However, the HGWO outlined in this paper surpasses all algorithms in terms of solution precision and attains the lowest fabrication expenditure.

**Table 10:** Experimental results on WBD.

Algorithm	$h(x_{1} )$	$l(x_{2} )$	$t(x_{3} )$	$b(x_{4} )$	Cost vaule
HGWO	0.205549486	3.4745588	9.036164628	0.205750555	**1.725201706**
GWO	0.205716787	3.474234074	9.03732048	0.205804924	1.72603458
WOA	0.204983931	3.514694073	9.131330514	0.205261886	1.74250256
MVO	0.200968323	3.581143328	9.040933223	0.205865208	1.734049104
SSA	0.199162521	3.617308325	9.03747941	0.20572539	1.734339988
AOA	0.215113287	3.199832005	10	0.216478387	1.954896205
SOA	0.20148161	3.72306601	8.66164218	0.23684058	1.916126993
GJO	0.20546688	3.477537666	9.038287721	0.206037492	1.728024723
AGWO_CS	0.199623864	3.623645813	9.037653905	0.206004339	1.738089007
PSOGWO	0.205324687	3.484019391	9.039536289	0.205718347	1.726472617
AGWO	0.205746644	3.489803969	9.01705171	0.20667157	1.731268551

Siginficant values are in bold.

### Three-bar truss design

**Figure 9:**
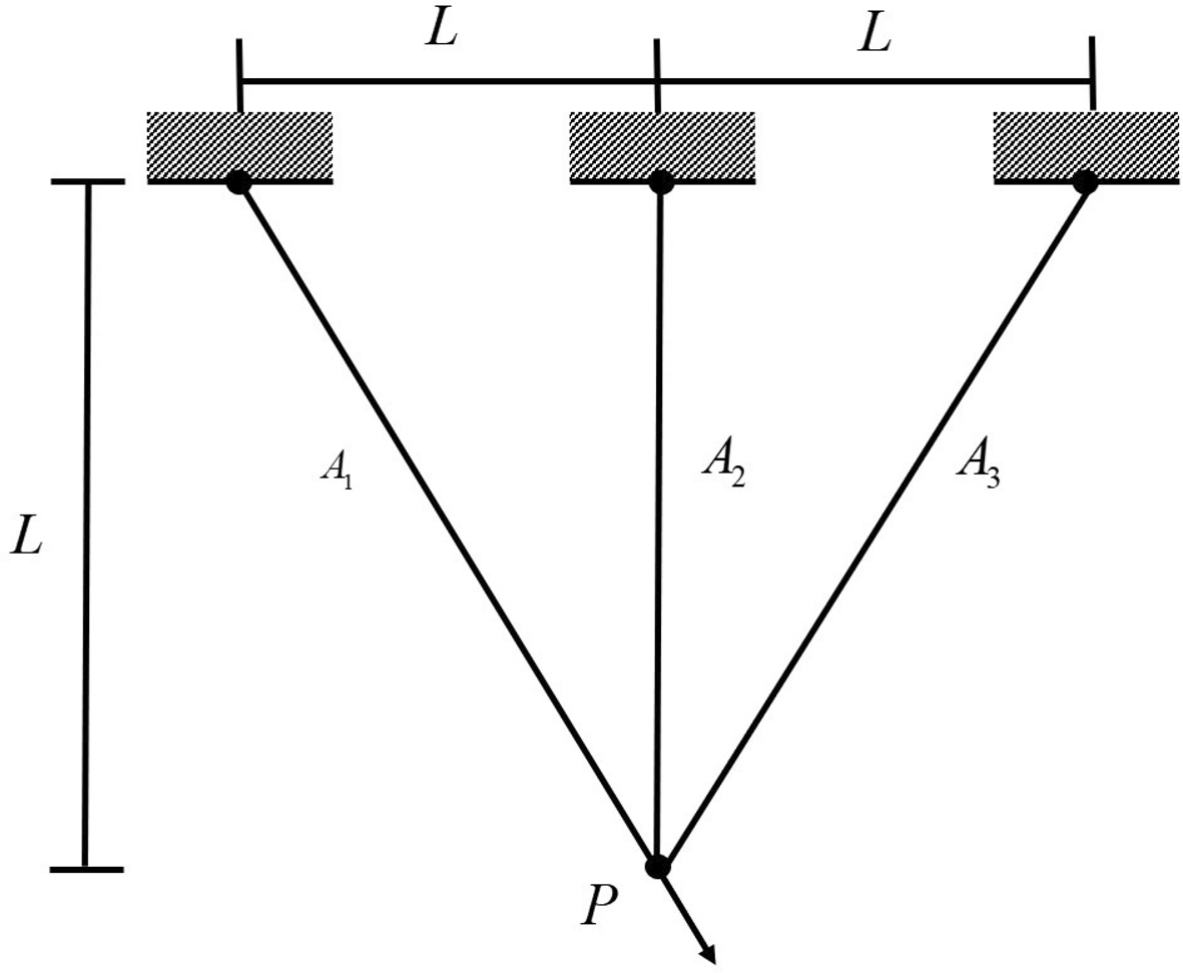
Three-bar truss design.

The design for a truss made of three-bar truss (TTD) is illustrated in Fig. 9. The main aim of this issue is to minimize the volume while satisfying the stress, deflection and curvature constraints on each side of the truss member^33^. Since the cross-sectional areas of the rods $A_{1} (x_{1} )$ and $A_{3} (x_{3} )$ in the three-bar truss are the same, only the cross-sectional areas of two rods $A_{1} (x_{1} )$ and $A_{2} (x_{2} )$ need to be selected as optimization variables.

Based on the results presented in Table 11, it is apparent that HGWO outperforms other methods in solving the three-bar truss design problem and achieves the lowest total cost.

**Table 11:** Experimental results on TTD.

Algorithm	$A_{1} (x_{1} )$	$A_{2} (x_{2} )$	Cost vaule
HGWO	0.788783438	0.407942067	**263.8958539**
GWO	0.789677227	0.405426093	263.897058
WOA	0.790859919	0.402103612	263.8993258
MVO	0.788838903	0.407787458	263.8960807
SSA	0.78840951	0.409000109	263.8958954
AOA	0.779683375	0.434796728	264.0074336
SOA	0.779790337	0.434068055	263.9648197
GJO	0.786157573	0.415425811	263.9015216
AGWO_CS	0.789155847	0.40704107	263.9110874
PSOGWO	0.789148302	0.406920744	263.8969208
AGWO	0.787173575	0.412558967	263.9022057

Siginficant values are in bold.

### Speed reducer design

**Figure 10:**
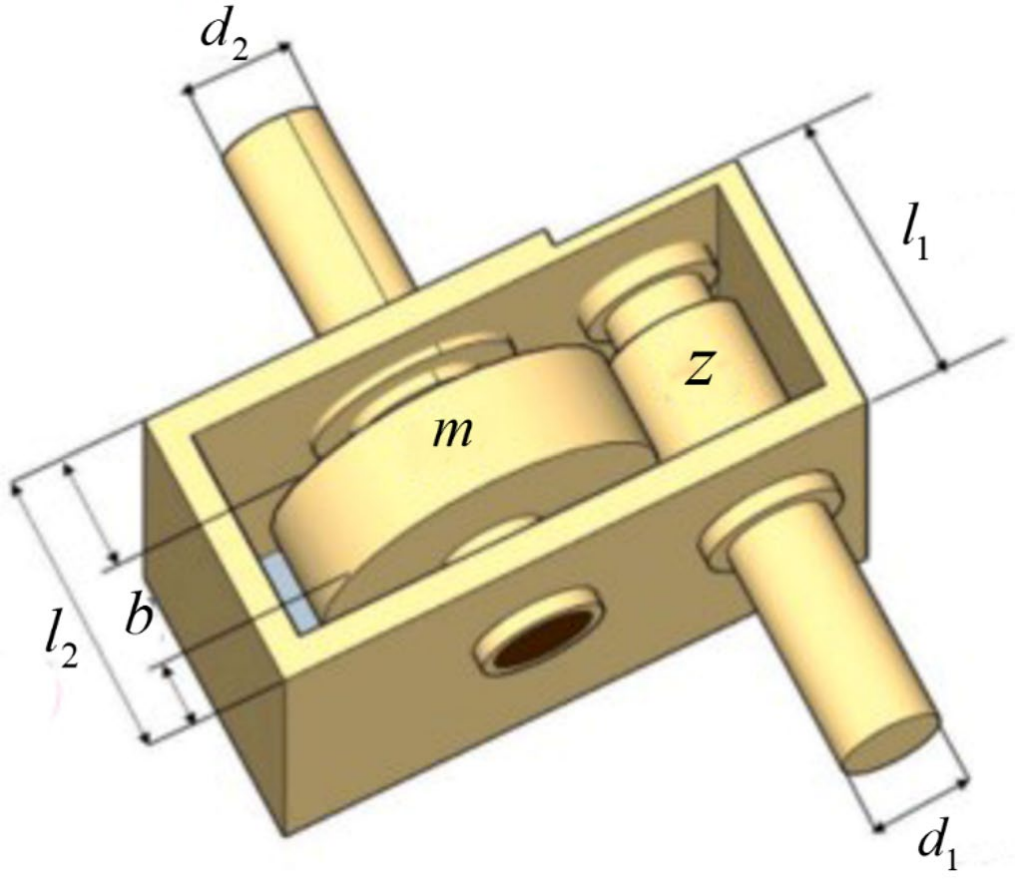
Speed reducer design.

Within mechanical systems, the reducer is a crucial element of the gearbox that is applied in several scenarios as indicated in Fig. 10. The objective of speed reducer design(SRD) problem is to minimize the weight of the gearbox while considering 11 constraints^87^. The problem has seven variables, which are the tooth face width $b(x_{1} )$, the gear module $m(x_{2} )$, the number of teeth in the pinion $z(x_{3} )$, the length of the first shaft between bearings $l_{1} (x_{4} )$, the length of the second shaft between bearings $l_{2} (x_{5} )$, the diameter of the first shaft of the bearings $d_{1} (x_{6} )$ and the diameter of the second shaft $d_{2} (x_{7} )$.

Table 12 illustrates the minimum cost and optimal solution of each algorithm utilized to solve the reducer design problem. Comparison of the solution outcomes of GWO, WOA, MVO, SSA, AOA, SOA, GJO, AGWO_CS, PSOGWO, and AGWO indicates that the solution of HGWO achieves the lowest fabrication cost.

**Table 12:** Experimental results on SRD.

Algorithm	$b(x_{1} )$	$m(x_{2} )$	$z(x_{3} )$	$l_{1} (x_{4} )$	$l_{2} (x_{5} )$	$d_{1} (x_{6} )$	$d_{2} (x_{7} )$	Cost vaule
HGWO	3.500079234	0.7	17	7.3	7.716117884	3.350542494	5.286665883	**2994.613691**
GWO	3.504543348	0.7	17	7.364205763	7.801421638	3.350412196	5.288055729	2999.653255
WOA	3.502539004	0.7	17	7.3	8.016778839	3.455646777	5.28675832	3029.866597
MVO	3.506707233	0.7	17	7.510353173	8.057684549	3.376851214	5.287039525	3013.590332
SSA	3.500049435	0.7	17	7.69459524	7.987871623	3.372677587	5.286748179	3009.820917
AOA	3.6	0.7	17	7.3	8.007455767	3.504896998	5.303088736	3091.742873
SOA	3.502169389	0.7	17	7.3	8.3	3.40308466	5.28731211	3022.263036
GJO	3.508009781	0.7	17	7.356993389	8.084522048	3.374382136	5.291056248	3015.243687
AGWO_CS	3.506377869	0.7	17.01557536	7.8	8.120712566	3.4	5.292996952	3030.0383
PSOGWO	3.500850651	0.7	17	7.360585956	7.943180207	3.367981355	5.293580331	3009.31643
AGWO	3.524711344	0.7	17	7.334452063	7.871395594	3.363760435	5.292441956	3015.057394

Significant values are in bold.

The findings suggest that the HGWO algorithm exhibits reduced production costs when compared to other algorithms in four engineering optimization problems of varying complexities. This highlights the effectiveness and applicability of the HGWO algorithm in engineering scenarios.

## Conclusions and future work

In this paper, a hybrid grey wolf optimizer (HGWO) is proposed, which incorporates the exploitation phase of the harris hawks optimization into the grey wolf optimizer for solving global optimization problems. Firstly, the LHS method is introduced in the population initialization phase to make the search agents more evenly distributed in the search space to improve the population diversity. Secondly, a new nonlinear convergence factor is constructed based on the properties of the sigmoid function, and a random number that conforms to the gamma distribution is added for perturbation to better balance the exploration and exploitation stages. To avoid the algorithm falling into local optimum during the iterative process, the influence of alpha wolves is weakened in the position update of the algorithm, and the interactions between individuals are increased and randomly selected with Levy flights, which greatly enhances the exploratory ability of the algorithm. Finally, in conjunction with the exploitation phase of the harris hawks optimization, the sharing of information about HHO, and the local search method, can promote the "wolves" in the GWO to better collaborate with each other and co-operate, thus speeding up the search process and increasing the diversity of searches. The idea of meritocracy based on greedy strategies is also used to further accelerate the convergence of the algorithm. Quantitative comparisons and convergence behavior analyses with a variety of standard optimization algorithms as well as different improved forms of grey wolf optimizer algorithms in 23 classical benchmark functions and CEC2020 show that the HGWO algorithm is able to obtain a better balance between global and local searches and significantly improves in terms of solution accuracy, convergence speed, and the ability to jump out of the local optimum. In addition, the algorithm has been applied to engineering problems such as pressure vessel design, welded beam design, three-bar truss design, and reducer design. The experimental results show that the improvement effect of HGWO is remarkable.

In future research, HGWO will be used for other real-world problems, including wireless network coverage, machine learning and logistics distribution. Furthermore, the low-parameter nature of the algorithm and its ability to be easily parallelized for simultaneous processing make it an attractive option for future applications on mobile devices, such as UAV trajectory planning.

### Supplementary Information

Supplementary Information.

## Data Availability

The datasets generated during and/or analysed during the current study are available from the corresponding author on reasonable request.
