# Supplementary material for: A hybrid algorithm of grey wolf optimizer and harris hawks optimization for solving global optimization problems with improved convergence performance
Source: Sci Rep. 2023 Dec 21;13:22909. doi: 10.1038/s41598-023-49754-2 (PMC10739963; doi:10.1038/s41598-023-49754-2)
Supplement: Supplementary file 1 — Supplementary Information. [file 41598_2023_49754_MOESM1_ESM.docx]

# Appendix

## *1 The process of GWO*

GWO improves optimization accuracy through more efficient search strategies, retains and updates optimal solutions, and maintains diversity and incremental evolution in the process.

### *1.1 Encircling prey*

$D=\left|C\cdot X_{p}(t)-X(t)\right|$(Ⅰ)

$X(t+1)=X_{p}(t)-A\cdot D$（Ⅱ）

Eq. (Ⅰ) computes the distance separating every grey wolf from the prey, and Eq. (Ⅱ) revises the position of each specific grey wolf. Where $X_{p}(t)$ and $X(t)$ represent the locations of the prey and the grey wolf respectively after the $t$ iteration. Random vectors $A$ and $C$ are calculated using Eqs. (Ⅲ) and (Ⅳ).

$A=2a\cdot r_{1}-a$（Ⅲ）

$C=2\cdot r_{2}$（Ⅳ）

$a=2(1-t/T)$（Ⅴ）

Where $r_{1}$ and $r_{2}$ represent random vectors in the interval [0, 1], while $T$ denotes the maximum number of iterations and $a$ signifies the linear convergence factor from 2 to 0.

### *1.2 Hunting prey*

Since the actual position of the prey is not known to the grey wolves during the whole iteration, the positions of $\alpha,\beta$ and $\delta$ wolves are directly assigned the prey's position after performing calculations, and the ω wolves update their positions accordingly, allowing the entire wolf pack to gradually approach and attack the prey.

$\begin{matrix} D_{\alpha}=\left|C_{1}\cdot X_{\alpha}(t)-X(t)\right| \\ D_{\beta}=\left|C_{2}\cdot X_{\beta}(t)-X(t)\right| \\ D_{\delta}=\left|C_{3}\cdot X_{\delta}(t)-X(t)\right| \end{matrix}$（Ⅵ）

$\begin{matrix} X_{1}=X_{\alpha}(t)-A_{1}\cdot D_{\alpha} \\ X_{2}=X_{\beta}(t)-A_{2}\cdot D_{\beta} \\ X_{3}=X_{\delta}(t)-A_{3}\cdot D_{\delta} \end{matrix}$（Ⅶ）

$X(t+1)=\left(X_{1}+X_{2}+X_{3}\right)/3$（Ⅷ）

Where $X_{\alpha}$,$X_{\beta}$ and$X_{\delta}$ denote the current positions of $\alpha,\beta$ and $\delta$wolves, respectively; $D_{\alpha}$, $D_{\beta}$ and $D_{\delta}$ denote the Euclidean distance between the search agent and $\alpha, \beta$,$\delta$wolves; $X_{1}$、$X_{2}$ and $X_{3}$ denote the position vectors of the search agent moving in the direction of $\alpha,\beta$ and $\delta$ wolves, respectively; Then the situation of the remaining wolves are determined according to Eq. (Ⅷ) after the iteration is updated to $X(t+1)$.

### *1.3 Attacking prey*

When the position of the prey is not changing, the grey wolf group then launches a fatal attack on the prey. The iterative process of the grey wolf approaching the prey is nonlinear, with the value of $a$ decreasing, resulting in a variation of $A$ within the range of [-2,2]. When the value of $A$ does not exceed the interval range, the position of the grey wolf after iteration can be arbitrary. When $\left|A\right|<1$, the grey wolves will attack their prey, indicating that the grey wolf group has reached a local optimum; when , the grey wolf will continue to explore other areas and find the global optimum.

## *2 Table Ⅰ Description of the 23 benchmark functions*

| **Function** | **Dim** | **Range** | | **Fmin** |
| --- | --- | --- | --- | --- |
| $f_{1}\left(x\right)=\sum_{i=1}^{n} x_{i}^{2}$ | 30 | [-100,100] | | 0 |
| $f_{2}\left(x\right)=\sum_{i=1}^{n} \left\vert x_{i}\right\vert+\prod_{i=1}^{n} \left\vert x_{i}\right\vert$ | 30 | [-10,10] | | 0 |
| $f_{3}\left(x\right)=\sum_{i=1}^{n} \left(\sum_{i=1}^{i} x_{i}^{2}\right)^{2}$ | 30 | [-100,100] | | 0 |
| $f_{4}\left(x\right)=\max\left\{\left\vert x_{i}\right\vert,1\leq i\leq n\right\}$ | 30 | [-100,100] | | 0 |
| $f_{5}\left(x\right)=\sum_{i=1}^{n} \left[100\left(x_{i+1}-x_{i}^{2}\right)^{2}+\left(x_{i}-1\right)^{2}\right]$ | 30 | [-30,30] | | 0 |
| $f_{6}\left(x\right)=\sum_{i=1}^{n} \left(\left\lfloor x_{i}+0.5\right\rfloor\right)^{2}$ | 30 | [-100,100] | | 0 |
| $f_{7}\left(x\right)=\sum_{i=1}^{n} ix_{i}^{4}+random[0,1)$ | 30 | [-1.28,1.28] | | 0 |
| $f_{8}\left(x\right)=\sum_{i=1}^{n} -x_{i}\sin\left(\sqrt{\left\vert x_{i}\right\vert}\right)$ | 30 | [-500,500] | -418.9829*Dim | |
| $f_{9}\left(x\right)=\sum_{i=1}^{n} \left[x_{i}^{2}-10\cos\left(2\pi x_{i}\right)+10\right]$ | 30 | [-5.12, 5.12] | | 0 |
| $f_{10}\left(x\right)=-20\exp\left(-0.2\sqrt{\frac{1}{n}\sum_{i=1}^{n} x_{i}^{2}}\right)-\exp\left(-\sqrt{\frac{1}{n}\sum_{i=1}^{n} \cos\left(2\pi x_{i}\right)}\right)+20+e$ | 30 | [-32,32] | | 0 |
| $f_{11}\left(x\right)=1+\frac{1}{400}\sum_{i=1}^{n} x_{i}^{2}-\prod_{i=1}^{n} \cos\left(\frac{x_{i}}{\sqrt{i+1}}\right)$ | 30 | [-600,600] | | 0 |
| $\begin{matrix} f_{12}(x)=\frac{\pi}{n}\left\{10\sin^{2}\left(\pi y_{i}\right)+\sum_{i=1}^{n-1} \left(y_{i}-1\right)^{2}\left[1+10\sin^{2}\left(\pi y_{i+1}\right)\right]\right. \\ \left.+\left(y_{n}-1\right)^{2}\right\}+\sum_{i=1}^{n} u\left(x_{i},10,100,4\right) \\ y_{i}=1+\frac{1}{4}\left(x_{i}+1\right) \\ u\left(x_{i},a,k,m\right)=\left\{\begin{matrix} k\left(x_{i}-a\right)^{m}, & x_{i}>a, \\ 0, & -a\leq x_{i}\leq a, \\ k\left(-x_{i}-a\right)^{m}, & x_{i}<-a. \end{matrix}\right. \end{matrix}$ | 30 | [-50,50] | | 0 |
| $\begin{matrix} f_{13}\left(x\right)=0.1\left\{\sin^{2}\left(3\pi x_{1}\right)+\sum_{i=1}^{n} \left(x_{i}-1\right)^{2}\left[1+\sin^{2}\left(3\pi x_{i}+1\right)\right]\right. \\ \left.+\left(x_{n}-1\right)\left[1+\sin^{2}\left(2\pi x_{n}\right)\right]\right\}+\sum_{i=1}^{n} u\left(x_{i},5,100,4\right) \end{matrix}$ | 30 | [-50,50] | | 0 |
| $f_{14}\left(x\right)=\left(\frac{1}{500}+\sum_{j=1}^{25} \frac{1}{j+\sum_{i=1}^{2} \left(x_{i}-a_{ij}\right)^{6}}\right)^{-1}$ | 2 | [-65.536,65.536] | | 1 |
| $f_{15}\left(x\right)=\sum_{i=1}^{11} \left[\left(a_{i}-\frac{x_{1}\left(b_{i}^{2}+b_{i}x_{2}\right)}{b_{i}^{2}+b_{i}x_{3}+x_{4}}\right)^{2}\right]$ | 4 | [-5,5] | | 0.0003075 |
| $f_{16}\left(x\right)=4x_{1}^{2}-2.1x_{1}^{4}+\frac{1}{3}x_{1}^{6}+x_{1}x_{2}-4x_{2}^{2}+4x_{2}^{4}$ | 2 | [-5,5] | | -1.3016285 |
| $f_{17}\left(x\right)=\left(x_{2}-\frac{5.1}{4\pi^{2}}x_{1}^{2}+\frac{5}{\pi}x_{1}-6\right)^{2}+10\left(1-\frac{1}{8\pi}\right)\cos x_{1}+10$ | 2 | [-5,10] [0,15] | | 0.3979 |
| $\begin{matrix} f_{18}\left(x\right)=\left[1+\left(x_{1}+x_{2}+1\right)^{2}\left(19-14x_{1}+3x_{1}^{2}-14x_{2}+6x_{1}x_{2}+3x_{2}^{2}\right)\right] \\ \times\left[30+\left(2x_{1}-3x_{2}\right)^{2}\left(18-32x_{1}+12x_{1}^{2}+48x_{2}-36x_{1}x_{2}+27x_{2}^{2}\right)\right] \end{matrix}$ | 2 | [-5,5] | | 3 |
| $f_{19}\left(x\right)=-\sum_{i=1}^{4} c_{i}\exp\left[-\sum_{j=1}^{4} a_{ij}\left(x_{j}-p_{ij}\right)^{2}\right]$ | 4 | [0,1] | | -3.86 |
| $f_{20}\left(x\right)=-\sum_{i=1}^{4} c_{i}\exp\left[-\sum_{j=1}^{6} a_{ij}\left(x_{j}-p_{ij}\right)^{2}\right]$ | 6 | [0,1] | | -3.32 |
|  | 4 | [0,10] | | -10.1532 |
|  | 4 | [0,10] | | -10.4029 |
|  | 4 | [0,10] | | -10.5364 |

## *3 Table Ⅱ Description of CEC2020*

| Type | No. | Functions | Fmin |
| --- | --- | --- | --- |
| Unimodal Functions | F24 | Shifted and Rotated Bent Cigar Function (CEC 2017 F1) | 100 |
| Basic Functions | F25 | Shifted and Rotated Schwefel's Function (CEC 2014 F11) | 1100 |
|  | F26 | Shifted and Rotated Lunacek bi-Rastrigin Function (CEC 2017 F7) | 700 |
|  | F27 | Expanded Rosenbrock's plus Griewangk's Function (CEC2017 F9) | 1900 |
| Hybrid Function | F28 | Hybrid Function 1 (N= 3) (CEC 2014 F17) | 1700 |
|  | F29 | Hybrid Function 1 (N= 4) (CEC 2014 F16) | 1600 |
|  | F30 | Hybrid Function 1 (N= 5) (CEC 2014 F21) | 2100 |
| Composition Functions | F31 | Composition Function 1 (N= 3) (CEC 2017 F22) | 2200 |
|  | F32 | Composition Function 1 (N= 4) (CEC 2017 F24) | 2400 |
|  | F33 | Composition Function 1 (N= 5) (CEC 2017 F25) | 2500 |
| Search range: [-100,100]^10^ | | | |

## *4 Mathematical description of the PVD problem:*

Objective function:

$$f(x)_{\min}=0.6224x_{1}x_{3}x_{4}+1.7781x_{2}x_{3}^{2}+3.1661x_{1}^{2}x_{4}+19.84x_{1}^{2}x_{3}$$

Subject to:

$$\begin{matrix} g_{1}(x)=-x_{1}+0.0193x_{3}\leq0 \\ g_{2}(x)=-x_{2}+0.00954x_{3}\leq0 \\ g_{3}(x)=-\pi x_{3}^{2}x_{4}-\frac{4}{3}\pi x_{3}^{3}+1296000\leq0 \\ g_{4}(x)=x_{4}-240\leq0 \end{matrix}$$

Variable range:

$$\begin{matrix} 0\leq x_{i}\leq100 i=1,2 \\ 10\leq x_{j}\leq200 j=3,4 \end{matrix}$$

## *5 Mathematical description of the WBD problem:*

Objective function:

$$f(x)_{\min}=1.10471x_{1}^{2}x_{2}+0.04811x_{3}x_{4}\left(14.0+x_{2}\right)$$

Subject to:

$$\begin{matrix} \text{g}_{1}(x)=\tau(x)-\tau_{\max}\leq0 \\ \text{g}_{2}(x)=\sigma(x)-\sigma_{\max}\leq0 \\ g_{3}(x)=\delta(x)-\delta_{\max}\leq0 \\ \text{g}_{4}(x)=x_{1}-x_{4}\leq0 \\ \text{g}_{5}(x)=\text{P}-\text{P}_{\text{c}}(x)\leq0 \\ \text{g}_{6}(x)=0.125-x_{1}\leq0 \\ \text{g}_{7}(x)=1.10471x_{1}^{2}+0.04811x_{3}x_{4}\left(14.0+x_{2}\right)-5.0\leq0 \end{matrix}$$

Variable range:

$$\begin{matrix} 0.1\leq x_{1}\leq2 \\ 0.1\leq x_{2}\leq10 \\ 0.1\leq x_{3}\leq10 \\ 0.1\leq x_{4}\leq2 \end{matrix}$$

Where,$\sigma_{\max}=30000psi$, $P=6000lb$，$L=14in$，$\delta_{\max}=0.25in$, $E=3\times10^{6}psi$,$\tau_{\max}=16000psi$, $G=1.2\times10^{7}psi$; the equation below can be used to reference the expression of each function in the constraint.

$$\begin{matrix} \tau(x)=\sqrt{\tau^{'2}+2\tau'\tau''\left(x_{2}/\text{R}\right)+\left(\tau''\right)^{2}} \\ \tau'=\frac{\text{P}}{\sqrt{2}x_{1}x_{2}},\tau''=\text{MR}/\text{J},\text{M}=\text{p}\left(\text{L}+x_{2}/2\right) \\ \text{R}=\sqrt{\frac{\left[x_{2}^{2}+\left(x_{1}+x_{3}\right)^{2}\right]}{4}} \\ \text{J}=2\left\{\sqrt{2}x_{1}x_{2}\left[\frac{x_{2}^{2}}{12}+\frac{\left(x_{1}+x_{3}\right)^{2}}{14}\right]\right\} \\ \text{P}_{\text{c}}(x)=\frac{4.013\text{E}x_{3}x_{4}^{2}}{6\text{L}^{2}}\left(1-\frac{x_{3}\sqrt{\text{E}}}{8\text{LG}}\right) \end{matrix}$$

## *6 Mathematical description of the TTD problem:*

Objective function:

$$f(x)_{\min}=\left(2\sqrt{2}x_{1}+x_{2}\right)\cdot L$$

Subject to:

$$\begin{matrix} \text{g}_{1}(x)=\frac{\sqrt{2}x_{1}+x_{2}}{\sqrt{2}x_{1}^{2}+2x_{1}x_{2}}P-\sigma\leq0 \\ \text{g}_{2}(x)=\frac{x_{2}}{\sqrt{2}x_{1}^{2}+2x_{1}x_{2}}P-\sigma\leq0 \\ \text{g}_{3}(x)=\frac{1}{\sqrt{2}x_{2}+x_{1}}P-\sigma\leq0 \end{matrix}$$

Variable range:

$0\leq x_{i}\leq1 i=1,2$（45）

Where, $L=100cm$，$P=2kN/cm^{2}$，$\sigma=2kN/cm^{2}$

## *7 Mathematical description of the SRD problem:*

Objective function:

$$\begin{matrix} f(x)_{\min}=0.7854\text{x}_{1}\text{x}_{2}^{2}\left(3.3333\text{x}_{3}^{2}+14.9334\text{x}_{3}-43.0934\right) \\ -1.508\text{x}_{1}\left(\text{x}_{6}^{2}+\text{x}_{7}^{2}\right)+7.4777\left(\text{x}_{6}^{3}+\text{x}_{7}^{3}\right)+0.7854\left(\text{x}_{4}\text{x}_{6}^{2}+\text{x}_{5}\text{x}_{7}^{2}\right) \end{matrix}$$

Subject to:

$$\begin{matrix} g_{1}(x)=\frac{27}{x_{1}x_{2}^{2}x_{3}}-1\leq0 \\ g_{2}(x)=\frac{397.5}{x_{1}x_{2}^{2}x_{3}^{2}}-1\leq0 \\ g_{3}(x)=\frac{1.93x_{4}^{3}}{x_{2}x_{6}^{4}x_{3}}-1\leq0 \\ g_{4}(x)=\frac{27}{x_{2}x_{7}^{4}x_{3}}-1\leq0 \\ g_{5}(x)=\frac{\sqrt{\left(745x_{4}/\left(x_{2}x_{3}\right)\right)^{2}+16.9\times10^{6}}}{110x_{6}^{3}}-1\leq0 \\ g_{6}(x)=\frac{\sqrt{\left(745x_{5}/\left(x_{2}x_{3}\right)\right)^{2}+157.5\times10^{6}}}{85x_{7}^{3}}-1\leq0 \\ g_{7}(x)=\frac{x_{2}x_{3}}{40}-1\leq0 \\ g_{8}(x)=\frac{5x_{2}}{x_{1}}-1\leq0 \\ g_{9}(x)=\frac{x_{1}}{12x_{2}}-1\leq0 \\ g_{10}(x)=\frac{1.5x_{6}+1.9}{x_{4}}-1\leq0 \\ g_{11}(x)=\frac{1.1x_{7}+1.9}{x_{5}}-1\leq0 \end{matrix}$$

Variable range:

$$\begin{matrix} 2.6\leq\text{x}_{1}\leq3.6 \\ 0.7\leq\text{x}_{2}\leq0.8 \\ 17\leq\text{x}_{3}\leq28 \\ 7.3\leq\text{x}_{4}\leq8.3 \\ 7.8\leq\text{x}_{5}\leq8.3 \\ 2.9\leq\text{x}_{6}\leq3.9 \\ 5.0\leq\text{x}_{7}\leq5.5 \end{matrix}$$
